# Supplementary material for: Low-methoxy pectin-containing enteral nutrition in critical care for intestinal tolerance (LOME-PECT): Study protocol for a randomized controlled trial
Source: PLoS One. 2025 Jul 11;20(7):e0326582. doi: 10.1371/journal.pone.0326582 (PMC12250234; doi:10.1371/journal.pone.0326582)
Supplement: S1 Table — (DOCX) [file pone.0326582.s001.docx]

Table 1. List of baseline data to be collected

| Category | Specific items |
| --- | --- |
| Background characteristics | Sex |
|  | Age |
|  | Height |
|  | Weight |
|  | Medical History |
|  | Cormobidities |
|  | Date of Consent obtained |
|  |  |
| Primary disease diagnosis |  |
|  |  |
| Disease category | infectious disease/ heart failure/ respiratory failure/ stroke/ renal or metabolic diseases/ postoperative/ post-cardioplumonary resuscitation/ trauma/ other |
|  |  |
| SOFA score |  |
|  |  |
| MUST | Weight loss in the past 3-6 months |
|  | Impaired nutritional intake for more than 5 days |
|  |  |
| Laboratory test | WBC |
|  | Lymphocyte count |
|  | CRP |
|  | Albumin |
|  | Prealbumin |
|  | Total cholesterol |
|  | HDL cholesterol |
|  | LDL cholesterol |
|  | Triglyceride |
|  |  |
| Gastrointestinal symptoms before initiation of EN | Presence or absence of diarrhea |
|  | Nausea |
|  | Vomiting |
|  | Abdominal pain |
|  | Intestinal ischemia |
|  | Ileus |
|  | Rectal bleeding |
|  |  |
| Gastric residual volume |  |
|  |  |
| Condition of stool | Bristol scale |

Abbreviations; SOFA, Sequential Organ Failure Assessment; MUST, Malnutrition Screening Tool; EN, enteral nutrition; WBC, white blood cell; CRP, C reactive protein; HDL, high-density lipoprotein; LDL, low-density lipoprotein
