# Supplementary material for: Low-methoxy pectin-containing enteral nutrition in critical care for intestinal tolerance (LOME-PECT): Study protocol for a randomized controlled trial
Source: PLoS One. 2025 Jul 11;20(7):e0326582. doi: 10.1371/journal.pone.0326582 (PMC12250234; doi:10.1371/journal.pone.0326582)
Supplement: S1 File — (DOCX) [file pone.0326582.s002.docx]

**Joint Clinical Research Agreement (investigator-initiated type)**

Yokohama City University and Otsuka Pharmaceutical Factory, Inc. have entered into a joint clinical research agreement with respect to the implementation of multicenter investigator-initiated joint clinical research as described in the following research implementation details, in consideration that the Research constitutes specific clinical research as defined in the Clinical Research Act (Act No. 16 of 2009, hereinafter referred to as the “Act”).

In view of the fact that this research falls under the category of specified clinical research as stipulated in the Clinical Research Act, the parties hereto have agreed to conclude an agreement stipulating the matters to be stated in Article 32 of the Act and Article 88 of the Enforcement Regulations of the Clinical Research Act (Ordinance of the Ministry of Health, Labor and Welfare No. 17 of 1991, including amendments after enforcement) and other notices and other matters applicable to this research.

The parties hereto have agreed to enter into an agreement as set forth below.

**【Research Implementation Details】**

1. Name of this study

| Study Name | Low-Methoxy Pectin containing Enteral nutrition in Critically care for intestinal Tolerance (LOME-PECT trial) |
| --- | --- |

1. Principal investigator(s) and affiliation(s)

| Name | Kensuke Nakamura |
| --- | --- |
| Affiliation (Representative Medical Institution) | Yokohama City University |
| Location of affiliated institution | 3-9 Fukuura, Kanazawa-ku, Yokohama, 236-0004 |
| Department | Department of Intensive Care, Yokohama City University Hospital |
| Name of the administrator | Itaru Endo (Hospital Director) |

1. The outline of the study
   1. Purpose, content and duration of this study

| Purpose | We evaluated the effect of a pectin-containing EN formula, HINEX® RENUTE on reducing intestinal intolerance (diarrhea) in patients admitted to the intensive care unit and receiving enteral nutrition (EN), using a pectin-free EN formula with similar energy density and three macronutrient balance as a control. |
| --- | --- |
| content | This is a multicenter, parallel-group, open-label, randomized controlled trial. Enrolled patients are those who require EN. Patients will receive either HINEX® RENUTE or GLUCERNA® REX via gastric tube for 3 days. The primary outcome is the incidence of diarrhea (as defined by Bristol Scale 5, 6, or 7) within the first 3 days of administration. Secondary outcomes include safety assessments and Bristol Scale on days 1, 2, and 3. |
| duration | From the following study start date to the study end date  ・Study start date: jRCT publication date  ・Study end date: the date on which publication is made by recording the Summary Report in the jRCT. |
| Target number of cases | 200 cases  (test group: 100 cases, control group: 100 cases) |
| Review Board | Yokohama City University Certified Institutional Review Board |

- 1. Outline of pharmaceuticals and other products to be used in this study

| type of drug, medical device, or regenerative medical product | ■Drug | □Medical device | □Regenerative medical product |
| --- | --- | --- | --- |
| Unapproved, off-label, and approved under the Pharmaceuticals and Medical Devices Act | ■Unapproved | □Off-label | □Approved |
| Generic name | Enteral formula | | |
| Brand name | HINEX® RENUTE | | |

1. The term of this Agreement and the funds for this research

| Contract Term of this Agreement | From the date of jRCT publication to March 31, 2029 |
| --- | --- |
| Funds for this research | Total amount: 18,181,200 JPY  (excluding consumption tax and local consumption tax) |

Translated by Shizuka Kashiwagi
