# Supplementary material for: Low-methoxy pectin-containing enteral nutrition in critical care for intestinal tolerance (LOME-PECT): Study protocol for a randomized controlled trial
Source: PLoS One. 2025 Jul 11;20(7):e0326582. doi: 10.1371/journal.pone.0326582 (PMC12250234; doi:10.1371/journal.pone.0326582)
Supplement: S2 File — (DOCX) [file pone.0326582.s003.docx]

Research Proposal

Title of Research Topic:

Intestinal intolerance of pectin-containing enteral nutritional preparations in critically ill patients

Validation of efficacy against: A multicenter, open-label, randomized controlled trial

LOME-PECT trial；Low-Methoxy Pectin containing Enteral nutrition in Critically care for intestinal Tolerance

**A number that identifies the study**

jRCTs031230684

Principal investigator: Kensuke Nakamura, Department of Intensive Care, Yokohama City University Hospital

Version Number: Version 2.0

Created on: June 18, 2024

Revision History

| Created Date | Number of Plates |
| --- | --- |
| January 15, 2024 | first edition |
| June 18, 2024 | Version 2.0 |
|  |  |
|  |  |

table of contents

[**0. Overview** 6](#_Toc169695667)

[0.1. Shema 6](#_Toc169695668)

[0.2. Aim of Study and Primary Endpoint 6](#_Toc169695669)

[0.3. Research Subjects 6](#_Toc169695670)

[0.4. Treatment 6](#_Toc169695671)

[0.5. Target number of cases 6](#_Toc169695672)

[0.6. Duration of the study 6](#_Toc169695673)

[0.7. Principal investigator and contact information 7](#_Toc169695674)

[**1. Purpose of this study** 8](#_Toc169695675)

[**2.** **Background and Scientific Basis of Study Design** 8](#_Toc169695676)

[2.1. Target Diseases 8](#_Toc169695677)

[2.2. Standard of Care 9](#_Toc169695678)

[2.3. Study Treatment 9](#_Toc169695679)

[2.4. Study Design and Primary Endpoint 9](#_Toc169695680)

[2.5. Significance of this research 9](#_Toc169695681)

[**3. Information on test products** 11](#_Toc169695682)

[3.1. Test Drugs, etc. 11](#_Toc169695683)

[3.2. Comparators, etc. 11](#_Toc169695684)

[3.3. Management of test products, etc. 11](#_Toc169695685)

[3.4. Ensuring the quality of test products, etc. 12](#_Toc169695686)

[**4. Criteria and definitions used in this study** 13](#_Toc169695687)

[**5. Selection Policy for Research Subjects** 14](#_Toc169695688)

[5.1. Selection criteria 14](#_Toc169695689)

[5.2. Exclusion Criteria 14](#_Toc169695690)

[**6. Research Plan** 15](#_Toc169695691)

[6.1. Study design 15](#_Toc169695692)

[6.2. Target Number of Cases 15](#_Toc169695693)

[6.3. Duration of the Study 15](#_Toc169695694)

[6.4. Facility Registration, Case Registration, and Allocation Methods 15](#_Toc169695695)

[6.5. Treatment Plan 16](#_Toc169695696)

[6.6. Discontinuation of the entire study 17](#_Toc169695697)

[**7. Observation, Inspection, Investigation, and Evaluation Items** 19](#_Toc169695698)

[7.1. Schedule 19](#_Toc169695699)

[7.2. Implementation Schedule and Evaluation Items 19](#_Toc169695700)

[**8. Evaluation items** 22](#_Toc169695701)

[8.1. Primary endpoint 22](#_Toc169695702)

[8.2. Secondary endpoint 22](#_Toc169695703)

[8.3. Safety endpoints 23](#_Toc169695704)

[9. Statistical Analysis 23](#_Toc169695705)

[9.1. Population to be analyzed 23](#_Toc169695706)

[9.2. Rationale for setting the target number of cases 23](#_Toc169695707)

[**9.3. Statistical Analysis Methods** 23](#_Toc169695708)

[**9.4. Interim Analysis** 24](#_Toc169695709)

[**9.5. General** 24](#_Toc169695710)

[**10. Handling of Diseases, etc.** 25](#_Toc169695711)

[10.1. Definition of Disease, etc. 25](#_Toc169695712)

[10.2. Evaluation of Diseases 25](#_Toc169695713)

[10.3. Foreseeable Diseases 26](#_Toc169695714)

[10.4. Measures to be taken in the event of illness 26](#_Toc169695715)

[10.5. Adverse Events Excluding Illness 27](#_Toc169695716)

[**11. DATA MANAGEMENT** 28](#_Toc169695717)

[**12. Effectiveness and Safety Evaluation Committee** 28](#_Toc169695718)

[**13. Management of compliance, modification and non-conformity with the research plan (deviation from the research plan, etc.)** 29](#_Toc169695719)

[13.1. Complying with the study plan 29](#_Toc169695720)

[13.2. Changes to the Research Proposal 29](#_Toc169695721)

[13.3. Management (Non-compliance with research plans, etc.) 29](#_Toc169695722)

[**14. Ethical Matters** 30](#_Toc169695723)

[14.1 Rules and Regulations to be complied 30](#_Toc169695724)

[14.2. Handling of Personal Information, etc. 30](#_Toc169695725)

[14.3. Expected benefits and disadvantages of research subjects as a result of research participation 30](#_Toc169695726)

[14.4. Handling of research results (including accidental findings) pertaining to research subjects 30](#_Toc169695727)

[14.5. Access to original documents 31](#_Toc169695728)

[**15. Procedures for Obtaining Informed Consent** 32](#_Toc169695729)

[15.1. Responding to Consultations from Research Subjects and Their Related Parties 32](#_Toc169695730)

[15.2. When obtaining informed consent from a substitute, etc. 33](#_Toc169695731)

[15.3. Obtaining Informed Ascent 33](#_Toc169695732)

[15.4. When you withdraw your consent after giving your consent 33](#_Toc169695733)

[15.5. When it is not necessary to obtain the consent of the specified clinical research subject, etc. 33](#_Toc169695734)

[**16. Methods of storage and disposal of samples and information** 34](#_Toc169695735)

[16.1. Secondary Use of Samples and Information 35](#_Toc169695736)

[16.2. Use of Samples and Information as a Biobank 35](#_Toc169695737)

[**17. Research on the management of the opposite of interests, etc** 35](#_Toc169695738)

[17.1. Sources and Financial Relationships 35](#_Toc169695739)

[17.2. Contrary Interests Management 35](#_Toc169695740)

[**18. Expenses and remuneration for research subjects** 36](#_Toc169695741)

[**19. Indemnity for Health Hazards** 36](#_Toc169695742)

[**20. Periodic Reporting** 36](#_Toc169695743)

[20.1. Periodic Reporting to the Accredited Clinical Research Review Board 36](#_Toc169695744)

[20.2. Periodic Report to the Minister of Health, Labour and Welfare 36](#_Toc169695745)

[**21. Disclosure of Research Information and Results** 37](#_Toc169695746)

[21.1. Registration of studies 37](#_Toc169695747)

[21.2. Publication of Research Results 37](#_Toc169695748)

[21.3. Publication of Academic Societies, etc. 37](#_Toc169695749)

[**22. Quality Management and Quality Guarantee** 38](#_Toc169695750)

[22.1. Monitoring 38](#_Toc169695751)

[22.2. Auditing 38](#_Toc169695752)

[**23. Attribution of Research Results (Intellectual Property Rights)** 38](#_Toc169695753)

[**24. Research Implementation Structure** 39](#_Toc169695754)

[24.1. Principal Investigator (or Principal Investigator) 39](#_Toc169695755)

[24.2. Research Secretariat 39](#_Toc169695756)

[24.3. Investigator and Investigator 39](#_Toc169695757)

[24.4. Data Management Officer 40](#_Toc169695758)

[24.5. Statistical Analyst 40](#_Toc169695759)

[24.6. Who is responsible for monitoring? 40](#_Toc169695760)

[24.7. Who is responsible for the audit? 40](#_Toc169695761)

[24.8. R&D Planning Officer 40](#_Toc169695762)

[24.9. Coordination Management Practitioners 40](#_Toc169695763)

[24.10. Persons other than the principal investigator who supervise the research 40](#_Toc169695764)

[24.11. Other clinical laboratories and medical and technical departments and institutions related to clinical research. 40](#_Toc169695765)

[24.12. Outsourced Organizations 40](#_Toc169695766)

[**25. References** 41](#_Toc169695767)

[**26. Appendix** 42](#_Toc169695768)

**Abbreviation table**

| acronym | Official name | explanation |
| --- | --- | --- |
| A-aDO2 | Alveolar-arterial Oxygen Difference | alveolar air, arterial blood oxygen partial pressure range |
| BSC | Best Supportive care | Treatments for symptom relief |
| Ca | Calcium | calcium |
| Cre | Creatinine | creatinine |
| CRP | C Reactive Protein | C-reactive protein |
| DHA | Docosahexaenoic Acid | Docosahexaenoic acid |
| DNR | Do Not Resuscitaion | Refusal of resuscitation |
| EDC | Electronic Data Capture | Electronic Data Acquisition Systems |
| IN | Enteral Nutrition | Enteral nutrition |
| EPA | Eicosapentaenoic Acid | Eicosapentaenoic acid |
| FiO2 | Fraction of inspiratory Oxygen | Inhaled oxygen concentration |
| G-CSF | Granulocyte-Colony Stimulating Factor | Granulocyte colonization stimulating factors |
| GCS | Glasgow Coma Scale | Glasgow Coma Scale |
| GRV | Gastric Residual Volume | Residual amount in the stomach |
| Hct | Hematocrit | Hematocrit |
| HDL | High Density Lipoprotein | High Specific Gravity Lipo Protein |
| ICU | Intensive Care Unit | Intensive Care Unit |
| jRCT | Japan Registry of Clinical Trials | Clinical Research Submission and Publication System |
| K | Potassium | potassium |
| LDL | Low Density Lipoprotein | Low-density lipoprotein |
| Mg | Magnesium | magnesium |
| On | Sodium | sodium |
| PaO2 | Partial pressure of arterial Oxygen | Arterial oxygen partial pressure |
| PEG | Percutaneous Endoscopic Gastrostomy | (percutaneously augmented) gastric wax |
| PTEG | Percutaneous Trans-Esophageal Gastro-tubing | Gastric tube (inserted percutaneously through the cervical esophagus) |
| RCT | Randomized Control Trial | A comparative study of inaction |
| SOFA | Sequential Organ Fairure Assessment | Assessment score for multiple organ failure |
| TG | TolyGlyceride | Triglycerides |
| V | Vitamin | vitamin |
| WBC | White Blood Cell | leukocyte |

# **0. summary**

## 0.1. Shema

Randomized controlled trials

ICU patients aged 18 years and older who will receive enteral nutrition

explanation

Obtaining Consent

Registration, Random Assignment

Study treatment group

Enteral feeding with HINEX® RENUTE

: Administered for 3 days

Administered for a minimum of 3 days

Control group

Enteral nutrition with GLUCERNA® REX: Administered for 3 days

Primary endpoint:

Incidence of diarrhea within 3 days of administration of the test product (Bristol Scale 5-7)

## 0.2. Aim of Study and Primary Endpoints

Purpose:

In patients admitted to the intensive care unit (ICU) who undergo enteral nutrition (EN), the pectin-containing EN preparation HINEX® RENUTE has been shown to reduce intestinal intolerance (diarrhea) with pectin-containing EN preparations glucelna, which does not contain pectin and has a similar energy density and balance of the three major nutrients.Validate REX as a control.

Primary endpoint:

Incidence of diarrhea within 3 days of initiation of study article administration (assessed as Bristol Scale 5-7)

## 0.3. Study Subjects

Patients admitted to the ICU (severely ill) who require control with EN preparations

## 0.4. therapy

Control group: EN with GLUCERNA® REX (continuous transgastric administration)

Study treatment group: EN (continuous transgastric administration) with HINEX® RENUTE

## 0.5. Target number of cases

Control group: 100 cases

Study treatment group: 100 patients

## 0.6. Duration of the study

Research period: From the date of first publication of the jRCT (date of notification by the Minister of Health, Labour and Welfare) to March 31, 2028

Registration period: From the date of initial publication of the jRCT (date of notification by the Minister of Health, Labour and Welfare) to March 31, 2025

Planned period of participation of clinical study subjects: Participation period is up to 5 weeks from the date of consent acquisition (up to 1 day for screening, up to 35 days from the date of start of administration ~ until the last observation date, of which the administration period is up to 7 days)

## 0.7.　Principal investigator and contact information

Affiliation: Yokohama City University Hospital, Intensive Care Department

Name: Kensuke Nakamura

Contact: 045-787-2918

# **1. Purpose of this study**

For patients admitted to the intensive care unit (ICU) who undergo enteral nutrition (EN), the pectin-containing EN preparation Hynex Slignut has been shown to reduce intestinal intolerance (diarrhea) and the EN preparation glucerna without pectin, which has a similar energy density and balance of the three major nutrients. - Validate REX as a control.

# **2. Background and Scientific Basis of Study Design**

It has been suggested that EN is useful for administering adequate nutrition in the transgastrointestinal tract in critically ill patients (ICU patients), and there is evidence that initiation of EN within 24~48 hours reduces the incidence of infections1^).^ Based on this, international guidelines recommend the start of EN at an early stage2^)3)^ However, it is often difficult to continue EN due to complications4). In particular, diarrhea occurs in 15~50% of severely ill patients and is the most common cause of EN dose reduction or discontinuation. In addition, the absorption of EN is severely impaired during diarrhea5)-7), so the EN administered may be ineffective. Furthermore, the occurrence of diarrhea has been shown to be associated with worsening ICU mortality and prolongation of ICU stay and hospitalization. Therefore, its control is an important issue in EN management.

E2), which is caused by various factors in severe conditions, has been shown in several previous studies to be strongly affected by the selection of EN preparations, and it is necessary to appropriately select EN preparations that are less likely to cause diarrhea ^(E3,4)^.

Dietary fiber is a group of substances that are difficult to digest and contribute to the optimization of the water content of stool when it reaches the large intestine, so EN preparations containing water-soluble dietary fiber are expected to have a diarrhea suppression effect.8) Among them, pectin, which is a type of water-soluble dietary fiber, Basic studies have shown that small molecule pectin has the effect of suppressing diarrhea by adjusting the properties of stool in particular, as well as suppressing the rate of delivery of the drug to the small intestine and beyond due to the acidic environment in the stomach, and that small molecule pectin has the effect of suppressing diarrhea by adjusting the properties of stool.9) The effect of low-molecular-weight pectin-containing EN preparations on diarrhea suppression has been confirmed10).

Here, the test formulation, HINEX® RENUTE, is an EN formulation containing the above-mentioned small molecule pectin, and at the same time contains 50% lipid and 24% protein, achieving high nutrient density. Therefore, in this study, EN GLUCERNA® REX, which does not contain pectin, which has the closest nutrient density and three major nutrient balances, was used as a control against the test formulation HINEX® RENUTE. This study examined the antidiarrheal effect of pectin-containing EN preparations in RCTs.

## 2.1. Target Diseases

Patients admitted to the ICU (severely ill) who require control with EN preparations

[Rationale for setting]

Critically ill patients who require admission to the ICU often cannot be taken orally due to sedation, impaired consciousness, respiratory and circulatory failure, and are indicated for intravenous or transgastrointestinal artificial nutrition, but more physiological EN is considered to be preferable from the viewpoint of glycemic control and infection prevention.

However, at the same time, the control of diarrhea during EN in critically ill patients is an important issue because it causes abnormalities in gastrointestinal function and it is difficult to adequately administer EN due to side effects of EN, including diarrhea.

## 2.2. Standard of Care

2.2.1. History of standard treatment that has been implemented so far

There are various types of EN preparations, and they are not specifically specified in guidelines, etc., and there are no absolute regulations for their selection. International nutritional guidelines also state that the ratio of carbohydrates and lipids can be flexibly selected in severe conditions without significantly affecting the patient2).

2.2.2. Current Standard of Care

As mentioned above, there are no absolute regulations for the selection of EN preparations, but " GLUCERNA® REX " is one of the commonly used formulations because it has a nutrient density and balance close to the test formulation and has a standard composition.

## 2.3. Study Treatment

Pectin is a complex polysaccharide contained in the leaves, stems, and fruits of plants, and is a dietary fiber that is not broken down by digestive enzymes, and is also added to some commercial foods. Hynex Renut contains low-molecular-weight pectin and is expected to reduce diarrhea, which may be associated with infection complications and favorable discharge outcomes.

Pectin is a natural thickening stabilizer found in fruits and vegetables. It is widely used in foods such as jams and jellies. Since it passes through the body without being broken down in the digestive organs, it does not seem to have any side effects or harmful effects. Pectin is considered safe by organizations such as the European Food Safety Authority (EFSA) and the US Food and Drug Administration (FDA), and is also recognized as safe by the Ministry of Health, Labor and Welfare in Japan.

Pectin is also used in pharmaceuticals and cosmetics, and the medical risks associated with administration are estimated to be small.

## 2.4. Study Design and Primary Endpoint

Study design: Multicenter randomized controlled study

Primary endpoint: Incidence of diarrhea within 3 days of initiation of study product (Bristol Scale 5-7)

[Rationale for setting]

The suppression of diarrhea, which is an EN inhibitor, is a clinically important issue, and the effect of low-molecular-weight pectin-containing EN preparations will be evaluated prospectively

The Bristol Scale is used as an evaluation method for diarrhea11). This index is designed to classify human fecal morphology into seven categories, and is used as a research tool to evaluate the therapeutic effects of various intestinal diseases, as well as widely used in clinical evaluation.

## 2.5. Significance of this research

By clarifying the effect of low-molecular-weight pectin-containing EN preparations on diarrhea suppression in severely ill patients, it will be possible to consider low-molecular-molecular-weight pectin-containing EN preparations as candidates when selecting EN preparations, especially when there is a high risk of diarrhea or when diarrhea is already difficult to control. By selecting EN formulations based on the same concept in intensive care, we can present the idea of avoiding diarrhea and continuing EN, and we can present an approach to suppress diarrhea including guidelines.

# **3. Information on test products**

## 3.1. Test Drugs, etc.

Product name: HINEX® RENUTE

Test Item: HINEX® RENUTE

Ingredients: dextrin, porcine collagen peptide (including gelatin), medium-chain fatty acid triglyceride, vegetable oil, soybean protein enzyme degradation product (including soybean), yeast, algae-derived DHA/EPA-containing oil, carnitine, kelp extract/polysaccharide thickener, lysine, magnesium chloride, sodium pyrophosphate, leucine, V.C, potassium hydroxide, acidulant, calcium phosphate, isoleucine, valine, methionine, crystalline cellulose, histidine, phenylalanine, potassium pyrophosphate, threonine, tryptophan, V.B1, fragrance, V.E, niacin, iron pyrophosphate, calcium pantothenate, V.B6, v.b2, v.a, folic acid, V.K2, v.d, v.B12

Storage method: Store in a dark place, can be stored at room temperature, but store in a cold place as much as possible, avoid freezing places.

Manufacturing and sales company name: Otsuka Pharmaceutical Factory Co., Ltd.

Indications: N/A

Dosage (how to use): Concentrated liquid food (from enteral feeding tube, continuous administration or bolus administration several times a day)

Dosage form (appearance): Bag type (liquid)

Calorie concentration: 1 kcal/mL Osmotic pressure: approx. 380 mOsm/L Protein: 6.0 g/100 kcal Energy ratio: 24% protein, 26% carbohydrate, 50% fat

Dietary fiber 1.2g/100kcal of which pectin 0.68g/100kcal

## 3.2. Comparators, etc.

Product Name: GLUCERNA® REX

Test product name: GLUCERNA® REX

Ingredients: rapeseed oil, high oleic sunflower oil, indigestible dextrin, dextrin, isomaltulose, fructose, isosoybean protein, fructooligosaccharides, casein, Ca, oat fiber, sodium chloride, glucose, L-carnitine, medium chain fatty acid oil/casein, Na, glycerin, dietary lecithin, sodium citrate, potassium chloride, myo-inositol, calcium citrate, calcium carbonate, magnesium phosphate, magnesium chloride, potassium phosphate, carrageenan, gellan gum, V.C, potassium citrate, zinc gluconate, iron sulfide, V.E, niacin, copper gluconate, calcium pantothenate, V.A, V.B6, V.B1, V.B2, folic acid, biotin, V.D, V.B12

Storage method: direct sunlight, room temperature storage.

Manufacturer and sales company name: Abbott Japan G.K.

Indications: N/A

Usage and Dosage (How to use): Thick liquid food

Dosage form (appearance): Bag type (liquid)

Calorie concentration: 1 kcal/mL Osmotic pressure: approx. 560 mOsm/L Protein: 4.2 g/100 kcal Energy ratio: 17% protein, 33% carbohydrate, 50% fat

Dietary fiber 0.9g/100kcal

## 3.3. Management of test products, etc.

The principal investigator will give the "Test Product Management Procedure" to the person in charge of test product management at the medical institution to which the investigator belongs, and the person in charge of test product management will manage the test product appropriately according to the procedure manual.

## 3.4. Ensuring the quality of test products, etc.

Separately stipulated "Test Material Management Procedures" In addition, if the Principal Investigator determines that it is necessary to recall the test product for clinical research due to poor quality or other reasons, the Principal Investigator shall promptly report to the Accredited Clinical Research Review Board on the measures to be taken, such as suspension of clinical research. The principal investigator will make a decision on whether or not to continue the research based on the results of deliberations by the Accredited Clinical Research Review Committee, and notify the responsible physician at each medical institution.

1. Promptly instruct the investigators to discontinue the use of the test product and to withdraw it.
2. Prepare and store a collection record that describes the details of the collection, the results of the investigation of the cause, and the improvement measures.

# **4. Criteria and definitions used in this study**

Diarrhea ：Bristol scale12^)^

Type 1 Stool with a hard lump like a tree nut (rabbit feces)

Type 2 Short sausage-like lumpy stools (lump stools)

Type 3 sausage-like stool with cracks on the surface (rather hard),

Type 4 Sausages with a smooth and soft surface, or snake-like coils (normal stools)

Type 5 Soft half-solid stools with clear boundaries (soft stools)

Type 6 Porridge-like stools that are fluffy and soft with loosened boundaries (muddy stools)

Type 7 Watery stools without clumps (watery stools)

SOFA score13)

SOFA score = Respiratory System Points + Cardiovascular Points + Renal Points + Liver Points + Coagulation Points + Central Nervous System Points


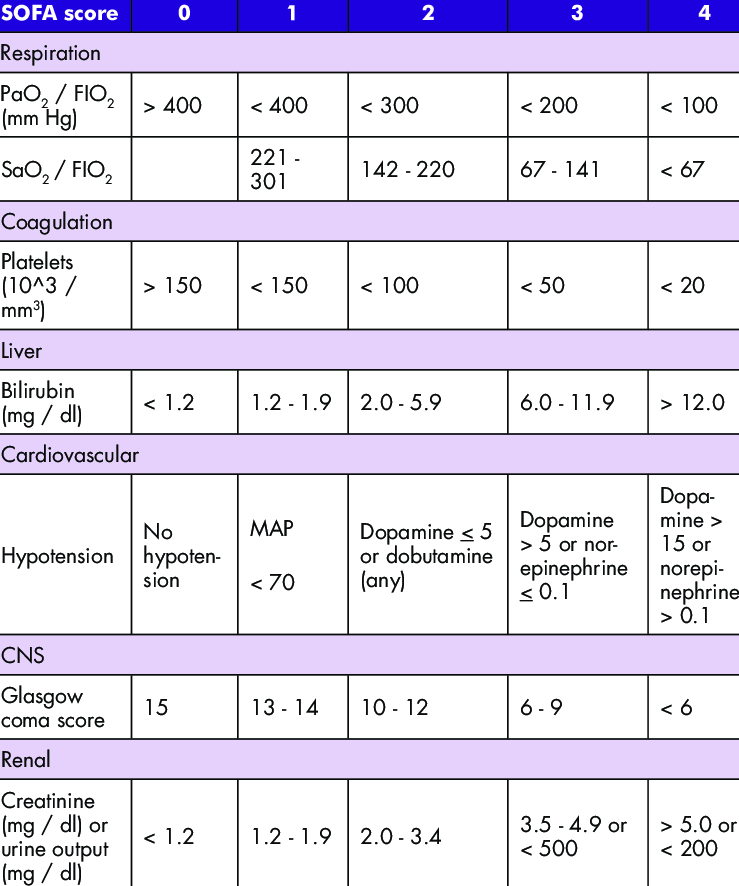


# **5. Selection Policy for Research Subjects**

## 5.1. Selection criteria

(1) Patients admitted to the ICU (regardless of the reason for admission)

(2) Patients who are 18 years of age or older at the time of enrollment

(3) Patients for whom EN by gastric administration is indicated

(4) Patients who have obtained written consent from the patient or their representative to participate in this study.

[Rationale for setting]

(1) This study was set up to investigate the effects of this study on severely ill patients

(2) This study was designed to target the legal age of adulthood

(3) There are two routes of EN administration: transgastric and transenteral, but only transgastric administration is intended to suppress diarrhea due to gelation of pectin in the stomach

(4) Established to protect the human rights and ensure the safety of research subjects

## 5.2. Exclusion Criteria

(1) Patients who had EN performed within 30 days prior to enrollment

(2) Patients with diarrhea at enrollment；Bristol scale≧5（Bristol scale 5，6，7点）

(3) Patients who are contraindicated or medically inappropriate (including allergies) for EN by gastric administration of this nutrient

(4) DNR/BSC policy patients

(5) Patients for whom EN is performed by PEG/PTEG

(6) In addition, when it is judged to be inappropriate by the judgment of a physician

[Rationale for setting]

(1) In the case of the most recent EN, the influence of other preparations cannot be ruled out, so it was set

(2) If diarrhea has already occurred, it is difficult to distinguish it from diarrhea caused by this preparation.

(3) It was established to ensure the safety of research subjects and to accurately evaluate safety and efficacy

(4) This study was given to patients with DNR/BSC because pain relief was given the highest priority, and the nutritional dosage and method were often different from the usual

(5) Administration from PEG/PTEG was exceptional, and the physical kinetics were different from administration from standard nasal feeding tubes

(6) This study was set up to include appropriate research subjects in this study

# **6. Research Plan**

## 6.1. Study design

This study is a multicenter, parallel-group, open-label, randomized controlled trial

## 6.2. Target Number of Cases

Number of cases of the total number of cases of the study: 200 cases (100 cases in Control group, 100 cases in Study treatment group)

For the basis for setting the number of cases, see "9.2 Rationale for setting the target number of cases"

## 6.3. Duration of the Study

Research period: From the date of initial publication of the jRCT (date of notification by the Minister of Health, Labour and Welfare) to March 31, 2028

Registration period: From the date of initial publication of the jRCT (date of notification by the Minister of Health, Labour and Welfare) to March 31, 2025

The participation period is up to 5 weeks from the date of obtaining consent (up to 1 day for screening, up to 35 days from the start date of administration ~ the date of last observation, of which the administration period is up to 7 days)

## 6.4. Facility Registration, Case Registration, and Allocation Methods

Facility registration and case registration will be based on the central registration system of the Registry Center (department in charge: Intensive Care Department, Yokohama City University Hospital).

6.4.1. Registration Center

Contact Person: Kensuke Nakamura

Facility name: Yokohama City University Hospital Intensive Care Department

TEL：045-787-2800

Mail：nakamura.ken.kl@yokohama-cu.ac.jp

Reception hours: Weekdays from 9 a.m. to 5 p.m. (not available on Saturdays, Sundays, national holidays, and year-end and New Year holidays)

6.4.2. Facility Registration

After obtaining approval from the Accredited Clinical Research Review Committee, the investigator of each medical institution sends a copy of the implementation approval form of the administrator of the implementing medical institution to the registration center.

6.4.3. Cases: Registered (and allocation).

(1) After obtaining written consent from the candidate research subject, the principal investigator or co-investigator shall conduct the tests necessary to determine eligibility.

(2) The principal investigator or co-investigator registers a case on the EDC, and at this time, each research institution assigns a research subject identification code, which is an arbitrary code that does not contain information that can identify a specific individual by itself.

(3) Eligibility will be determined on EDC, and patient registration and allocation will be performed.

(4) The principal investigator or co-investigator shall confirm the allocation results on the EDC immediately after registration.

(5) The principal investigator or co-investigator may view the results of the randomization at any time on the EDC.

6.4.4. Cutting MethodとCutting Adjustment Factor

Subjects are randomly assigned on the EDC for enrollment, and block randomization is performed using a program in the EDC (created on behalf of TXP Medical Co., Ltd.).

6.4.5. Methods of Blinding

Not applicable because we are not blinded in this study.

6.4.6. Determination and Procedure for Necessity of Key Opening

Not applicable because we are not blinded in this study.

## 6.5. Treatment Plan

6.5.1. Protocol Treatment

6.5.1.1. Study treatment group

HINEX® RENUTE is administered continuously through a feeding tube through the stomach for 3 days.

6.5.1.2. Control treatment group

GLUCERNA® REX is administered continuously through a feeding tube through the stomach and continues for 3 days.In addition, under the judgment of the attending physician, the drug can be continued until the 7th day, but the test preparation is not administered.

There is no limit to the period from enrollment to the start of administration of the test product, and EN will be started at a time judged to be medically appropriate. As a nutritional protocol for both groups, the dose rate will start at 10 ml/h, and if there are no adverse events, the dose will be increased by 10 ml/h every 8 hours. The target energy dosage can be determined by the attending physician without prescribing.

[Rationale] E5 with reference to the rate of administration commonly introduced in protocols for enteral nutrition in severe conditions)

6.5.2. Dosage Criteria

6.5.2.1. Initiation Criteria

It shall be at the discretion of the physician in charge, and no special provisions shall be made.

6.5.2.2 Weight Reduction and Discontinuation Criteria

In the following cases, consider postponing, decreasing, or discontinuing the dose of the dose (as appropriate by the attending physician).

(1) In the case of 200 ml/8 h or more of residual gastric fluid
(refer to the record of aspiration and contents of the nasogastric tube by the nurse in charge as a normal medical treatment)

(2) If diarrhea occurs

(3) In addition, when adverse events due to EN are recognized and the attending physician deems the dose increase inappropriate.

6.5.2.3 Resume Criteria

We do not set a resumption criterion because we do not set a discontinuation standard.

6.5.3. Concomitant medications and therapies

- - The item name "XX treatment group" should be appropriately corrected, such as changing it to the actual drug name.
  - Prescribe how many days after enrollment you need to start administering the study treatment.
  - Specify the dosage, dose, and duration of administration of the test treatment.
  - Describe the items to be complied with (e.g., length of hospitalization if hospitalization is required).

6.5.3.1. Concomitant prohibited treatment

Enteral nutritional preparations other than protocol treatment will not be used in both groups until the primary outcome assessment, but non-nutritional drugs can be administered, intravenous nutrition is allowed as appropriate, and there are no restrictions on other treatments.

6.5.3.2. Concomitant caution treatment

Not applicable

6.5.3.3. Concomitant treatment

(1) G-CSF and antibiotics for febrile neutropenia and other infectious diseases

(2) Prophylactic 5HT3 antagonists for the reduction of nausea and vomiting

(3) Antidiarrheal agent

(4) Topical steroids for skin symptoms

(5) Opioids use for analgesic purpose

(6) Other symptomatic treatment for complications and adverse events

6.5.4. Discontinuation of Studies in Individual Study Subjects

6.5.4.1. Discontinuation Criteria

If any of the following criteria are met, the study of the study subject will be discontinued.

(1) When there is a request for withdrawal of consent from the patient or the representative

(2) If it is found that the applicant does not meet the selection criteria or violates the exclusion criteria after registration, and is inappropriate as a target.

(3) When it is difficult to continue research due to deterioration of symptoms or findings of the disease

(4) When it is difficult to continue the study due to the occurrence of adverse events

(5) In the event of a significant deviation from the research plan

(6) In addition, when the principal investigator or co-investigator determines that the continuation of the research is not desirable

6.5.4.2. Discontinuation Procedure

(1) In the event that the principal investigator or co-investigator discontinues the administration of the test product to the research subject during the research period, the principal investigator or co-investigator shall promptly explain the discontinuation to the research subject and make necessary observations, examinations, and evaluations.

(2) In the event that the principal investigator or co-investigator discontinues administration of the test product to the study subject due to safety issues of the research subject, such as worsening of symptoms and the occurrence of adverse events, the principal investigator or co-investigator will take appropriate measures against the study subject.

(3) The date and reason for discontinuation of administration should be stated in the case report form.

6.5.4.3. Post-treatment after end of protocol treatment (discontinuation)

Not applicable in this study.

## 6.6. Discontinuation of the entire study

In the event that the study is discontinued, the investigator shall promptly notify the research subject of the discontinuation and take other necessary measures such as providing appropriate medical care. Prepare a Uniform Form 11 Discontinuation Notice and notify the Accredited Clinical Research Review Board. In addition, the principal investigator shall prepare Form 4 Notification of Discontinuation of Specific Clinical Research. Even if the research is discontinued, the primary endpoint report, the summary report, and the summary thereof shall be prepared appropriately, and periodic reports and disease reports will be conducted until the preparation and completion of the research.

(1) When an unpredictable serious illness occurs, and there is a concern that it will be detrimental to the entire research subject.

(2) When it is judged that there is no efficacy or superiority based on the results of interim analysis, etc.

(3) When a serious violation or non-conformity with the law, related laws and regulations, or the research plan is found

(4) When facts that impair or may impair ethical validity or scientific rationality are obtained.

(5) When a significant risk to the research subject is identified.

(6) When it is judged that it is difficult to complete the study due to reasons such as significant delays in patient registration or frequent deviations from the research protocol.

(7) When a discontinuation request or recommendation is received from the administrator of the implementing medical institution or the Accredited Clinical Research Review Board.

(8) When the Minister of Health, Labour and Welfare receives a cancellation request or recommendation.

# **7. Observation, Inspection, Survey, and Evaluation Items**

## 7.1. Schedule

| enforcement | | screening | administration | Duration of administration | | | Post-observation | | At Exit | At the time of cancellation |
| --- | --- | --- | --- | --- | --- | --- | --- | --- | --- | --- |
|  | | period | start date  Day0 | Week 0  Day1 | Week 0  Day2 | Week 0  Day3 | 1 week  Day7 | 2 weeks  Day14 | 4 weeks  D28 |  |
| Tolerance (days) | | ICU admission~0 | 0 | 0 | 0 | 0 | ±2 | ±2 | ＋7 | ±3 |
| Obtaining Consent and Registration | | ○ |  |  |  |  |  |  |  |  |
| Patient Background Verification | | ○ |  |  |  |  |  |  |  |  |
| Dosing of test products | |  |  | 🡨--------------------🡪 | | | Note 1 |  |  |  |
| SOFA Score | | ○ |  |  |  |  |  |  |  |  |
| Nutrition Assessment Information | | ○ |  |  |  |  |  |  |  |  |
| Gastrointestinal symptoms | |  | ○ | ○ | ○ | ○ |  |  |  |  |
| Bowel movement findings | |  | ○ | ○ | ○ | ○ |  |  |  |  |
| Confirmation of laxative administration | |  | ○ | ○ | ○ | ○ |  |  |  |  |
| Blood preservation Note 2 | |  | ○ |  |  | ○ | ○ |  |  |  |
| Preservation of stool Note 2 | |  | ○ |  |  | ○ | ○ |  |  |  |
| Pro  bed  check  investigate | Hematology  inspection | ○ | ○ | ○ |  | ○ | ○ | ○ |  | ○ |
|  | Blood biochemistry  inspection | ○ |  |  |  |  | ○ | ○ |  | ○ |
| Death Confirmation | |  |  |  |  |  |  |  | ○ |  |
| ICU Admission Period Information | |  |  |  |  |  |  |  | ○ |  |
| Observation of adverse events | |  | 🡨------------------------------------------------------------🡪 | | | | | | | ○ |

1. Can be administered after Day 3 of the administration period under clinical judgment
2. Only for subjects who have obtained consent as an option

## 7.2. Implementation Schedule and Evaluation Items

7.2.1. Screening Period

1. Background of Research Subjects

- Gender, age, height, weight, medical history, complications
- Date of Consent Acquisition
- Name of diagnosis of the primary disease, disease category (infection, heart failure, respiratory failure, stroke, renal and metabolic disease, postoperative, CPA resuscitation, trauma, other)

1. SOFA Score

- Respiratory points
- New Vascular System Points
- Renal system points
- Hepatic system points
- Coagulation system points
- Central nervous system points

1. Nutritional Assessment Information (MUST)
2. Hematological test (WBC, lymphocyte count)
3. Blood biochemical tests (CRP, albumin, prealbumin, gross/HDL/LDL cholesterol, triglycerides)

7.2.2. 0 days (before the start of administration)

1. Gastrointestinal symptoms (nausea/vomiting, abdominal pain, mean 3-day GRV/8h, intestinal ischemic event, ileus findings, presence or absence of melena)
2. The presence or absence of diarrhea on the Bristol Scale, (200 g/day, or 300ml/day), or (water-soluble diarrhea more than 3 times a day) will
   be evaluated by the nurse in charge at the time of excretion treatment and recorded in the medical record (the same shall apply on days 1~3)
3. Confirmation of the presence or absence of laxatives
4. Hematological test (WBC, lymphocyte count)
5. Adverse events
6. Plasma and whole blood will be preserved in blood specimens on Day 0 (prior to intervention administration) (only for subjects with consent obtained as an option)
7. Preservation of feces on Day 0 (prior to administration of the intervention formulation) (only for subjects with consent obtained as an option)

7.2.3. 1 day (24 hours after the start of administration)

1. Gastrointestinal symptoms (nausea/vomiting, abdominal pain, mean 3-day GRV/8h, intestinal ischemic event, ileus findings, presence or absence of melena)
2. Presence or absence of diarrhea on the Bristol Scale, (200 g/day, or 300 ml/day), or (water-soluble diarrhea more than 3 times a day)
3. Confirmation of the presence or absence of laxatives
4. Hematological test (WBC, lymphocyte count)
5. Adverse events

7.2.4. 2 days (48 hours after start of administration)

1. Gastrointestinal symptoms (nausea/vomiting, abdominal pain, mean 3-day GRV/8h, intestinal ischemic event, ileus findings, presence or absence of melena)
2. Presence or absence of diarrhea on the Bristol Scale, (200 g/day, or 300 ml/day), or (water-soluble diarrhea more than 3 times a day)
3. Confirmation of the presence or absence of laxatives
4. Adverse events

7.2.5. 3 days (at 72 hours after initiation of administration)

1. Gastrointestinal symptoms (nausea/vomiting, abdominal pain, mean 3-day GRV/8h, intestinal ischemic event, ileus findings, presence or absence of melena)
2. Presence or absence of diarrhea on the Bristol Scale, (200 g/day, or 300 ml/day), or (water-soluble diarrhea more than 3 times a day)
3. Confirmation of the presence or absence of laxatives
4. Hematological test (WBC, lymphocyte count)
5. Adverse events
6. Preserve plasma and whole blood in blood specimens on Day 3 (only for subjects with optional consent)
7. Preservation of feces on Day 3 (optional for subjects with consent obtained)

7.2.6 Post-observation (1 week, 2 weeks)

1. Hematological test (WBC, lymphocyte count)
2. Blood biochemical tests (CRP, albumin, prealbumin, gross/HDL/LDL cholesterol, triglycerides)
3. Plasma and whole blood are preserved with blood samples on day 7 (± 1)
4. day7（±1）の糞便を保存
5. Adverse events

7.2.7. At the end of the week (4 weeks)

1. Death or absence 28 days after initiation of test product administration
2. ICU Admission Period Information

- Length of ICU days
- Length of in-hospital days
- Availability and duration of ventilator
- Use of hemodialysis
- Use of extracorporeal membrane oxygenation (ECMO)
- Whether or not a vasopressor was administered (assuming that any one of the vasopressor was used as a vasopressor is dopamine, dobutamine, norepinephrine, epinephrine, or vasopressin) and
   whether or not the drug was administered within 3 days after administration of the test product (specific drug names are not collected)
- Presence or absence of continuous sedation (assuming that one of the sedatives is propofol, midazolam, dexmedetomidine, thiopental, or ketamine) and
  whether or not the drug was administered within 3 days after administration of the test product (specific drug names are not collected)
- Continuous opioid administration (assuming that any one of fentanyl, remifentanil, or morphine is used as an opioid) and
  whether or not the drug was administered within 3 days after administration of the test product (specific drug names are not collected)
- Collect whether or not a drug to improve gastrointestinal function is administered within
   3 days after administration of the test product (specific drug names are not collected)
- Barthel Index at Day 28 after the start of study product administration (Barthel Index at the time of discharge if discharged from the hospital by then)
- Presence or absence of EN failure at days 3 and 7 after initiation of EN administration (if any, selection of reasons: diarrhea, vomiting, intestinal ischemia, other EN adverse events, extubation, initiation of meals, examination/room transfer, other reasons for non-EN adverse events)
- EN Duration of Administration
- Daily energy dose kcal/day, protein dose g/day divided into EN and intravenous nutrition for the first 7 days after starting EN
- Presence or absence of use of prohibited drugs

1. Adverse events

7.2.8. Cancellation

1. Hematological test (WBC, lymphocyte count)
2. Blood biochemical tests (CRP, albumin, prealbumin, gross/HDL/LDL cholesterol, triglycerides)
3. Adverse events

# **8. Evaluation items**

## 8.1. Primary endpoint

Incidence of diarrhea (as defined by Bristol Scale 5, 6 and 7) within 3 days of the start of study article administration

(If diarrhea is confirmed even once during the above period, an event will occur.)

(6.5.2.2 Even if the dose is reduced or discontinued in accordance with the Weight Reduction and Discontinuation Standards, an event will occur if the definition is satisfied.)

[Rationale for setting] Diarrhea at the time of EN is often difficult to control, and the presence or absence of diarrhea is a matter of high interest.

Since the intestinal residence time of ordinary EN preparations is more than 24 hours for lipids that take the longest time to absorb, it is considered that it takes 24 to 48 hours for EN to penetrate the entire intestinal tract after EN is started, and diarrhea associated with EN preparations can occur within 24 hours thereafter, and it is considered sufficient to determine the occurrence of diarrhea within 3 days of starting EN preparations.

The Bristol Scale is used as an indicator of diarrhea based on citations in past studies and recommendations in recent reviews11), and diarrhea is defined as a scale of 5 or higher that has a significant clinical effect.

## 8.2. Secondary endpoint

・Presence or absence of diarrhea on the Bristol Scale, (200 g/day, or 300 ml/day) on the 1st, 2nd, or 3rd day of administration of the test product, or presence or absence of water-soluble diarrhea (Bristol Scale 7) more than 3 times a day

- Occurrence of diarrhea (as defined in Bristol Scale 5, 6, and 7) during the first week after the start of administration of the test product, (supplemented by medical record review)

・EN failure rate on days 3 and 7 of the start of study product: Percentage of subjects who failed EN due to diarrhea, vomiting, intestinal ischemia, or other reasons for adverse EN events (extubation, initiation of meals, examination/room transfer, and other reasons that are not EN adverse events are not included, even if they fail EN)

・Duration of EN administration

・For the first 7 days after the start of administration of the test product, the daily energy dose kcal/day, protein dose g/day, and protein dose g/day, and the energy amount does not include intravenous nutrition and propofol nutrition with an energy density thinner than 5% glucose solution.

・Survival rate of 28 days after start of administration of the test product

- Number of days in the ICU

・Number of days of hospitalization

・Number of days of artificial respiration

・Nutritional endpoints: WBC, lymphocyte count, CRP, albumin, prealbumin, gross/HDL/LDL cholesterol level, triglyceride level (day 7, day 14)

・Barthel Index at day 28 after the start of administration of the test product (if the patient is discharged from the hospital by then, it will be the Barthel Index at the time of discharge)

## 8.3. Safety endpoints

Adverse events and adverse reactions will be collected as safety endpoints.

Incidence of intestinal intolerance events

: Gastrointestinal symptoms (nausea/vomiting, abdominal pain, mean of 3-day GRV/8h, intestinal ischemic event, ileus findings, presence or absence of melena)

Definition of intestinal ischemia: one of the following (when imaging tests such as CT suggest hemostasis of the celiac artery, superior mesenteric artery, or inferior mesenteric artery, when colon ischemia is found by endoscopy, or when intestinal ischemia is found on surgical findings)^15)^

Incidence of infection events within 28 days of initiation of test product administration: nosocomial pneumonia, bacteremia, catheter-associated infections, urinary tract infections, soft tissue infections, and other infections

Definition of nosocomial pneumonia: infiltrative shadow on chest radiograph and the following two (fever of >38°C, leukocyte count <4000 or >11000/μl, purulent sputum)^16)^

# 9. Statistical Analysis

## 9.1. Population to be analyzed

The target population is defined as follows: the main target population is FAS, and the analysis of adverse events is SAS.

9.1.1. Full analysis set：FAS

FAS is defined as a population that has undergone at least one trial treatment and has been observed for primary outcomes.

9.1.2. Safety analysis set：SAS

SAS is defined as the population enrolled in this study and received at least one dose of the test product.

## 9.2. Rationale for setting the target number of cases

The total number of cases studied: 200 (100 cases in study treatment group and 100 cases in the control group).

[Rationale for setting]

The incidence of diarrhea in each group of this study was estimated using data from past retrospective studies (10) as a confirmation test. The diarrhea in the control group (GLUCERNA) was estimated to be 50%, about 10% higher than that of traditional liquid EN (May Balance) in the previous study, because it contained more lipids. The diarrhea of the intervention group (HINEX® RENUTE) was estimated to be 30%, slightly higher than that of the previous study Pectin-containing liquid EN (which contains pectin but contains less fat and is less concentrated than the previous study).

Assuming that the incidence of diarrhea in the intervention and control groups was 30% and 50%, and applying Pearson's chi-square test under a two-sided significance level of 5%, the number of patients required to maintain 80% power was calculated to be 93 in each group.

## **9.3. Statistical Analysis Methods**

**9.3.1. Analysis of Primary Endpoint**

**9.3.1.1 Key Analysis**

The following aggregation analysis is performed for FAS.

Frequency aggregation and confidence intervals (Wilson score intervals) are calculated, and comparisons between groups are performed using Pearson's chi-square test.

**9.3.1.2. Secondary analysis**

　The following subgroup analysis was performed on FAS.

・Gender (Male/Female)

・Age (under 70 years old/over 70 years old)

・BMI (20 or more/20 or more, 30 or more/30 or more)

・ SOFA score (1 or less than 2 points in each item/other)

・Disease category (infectious disease, heart failure, respiratory failure, stroke, renal and metabolic diseases, postoperative / post-CPA resuscitation, trauma, other)

[Rationale] Since diarrhea is caused by many factors in critically ill patients, E2^),^ it is particularly useful to identify subgroups in which the test drug is effective and can be used for future practice

**9.3.2. Analysis of Secondary Endpoints**

The following aggregation analysis is performed for FAS. We also calculate the confidence interval (Pearson's Chi-Square) for the difference between groups.

**9.3.2.1. EN failure rate, 28-day survival rate after initiation of treatment**

Calculate frequency aggregation and confidence intervals (Wilson score intervals).

**9.3.2.2. Number of ICU admissions, hospitalizations, and mechanical ventilation days**

Calculate summary statistics.

**9.3.2.3. Other Secondary Endpoints**

Counts are aggregated for binary data, and summary statistics are calculated for continuous values.

Referring to the above aggregation results, we will consider subgroup analysis as a post-hoc analysis.

**9.3.3. Safety Analysis**

The following data is calculated for adverse events and adverse reactions for SAS.

The frequency of the number of occurrence cases is tabulated.In addition, the frequency of the number of incidence cases by event and severity is tabulated. In addition, the counts were counted for intestinal intolerance events and infection events within 28 days after the start of administration of the test product.

## **9.4. Interim Analysis**

In this study, no interim analysis was performed.

## **9.5. General**

- As a general rule, the significance level in the test is 5% for both sides, and the confidence interval is calculated as a 95% confidence interval for both sides
- Summary statistics for continuous variables are number of examples, mean, standard deviation, minimum, maximum, median, the first quartile and the third quartile are calculated.The number of decimal places entered in the case report form + 2 digits is rounded to the nearest + 1 digit. The maximum and minimum values are displayed in the same number of decimal places as entered in the case report form.
- Frequency aggregation for categorical variables calculates frequencies and proportions.The number of digits of the percentage is rounded to less than the first decimal place and the % is displayed to the first decimal place

# **10. Handling of Diseases, etc.**

## 10.1. Definition of Disease, etc.

Illness is a disease, disability, death, or infectious disease that is suspected to be caused by the conduct of a specific clinical study, including unintended signs, clinically significant fluctuations in laboratory values, symptoms, and worsening of complications.

In this study, events suspected of being caused by the implementation of specific clinical studies between the start date of treatment and the end of the post-observation period are treated as diseases, etc., and data on serious diseases and diseases of all grades are collected.

## 10.2. Evaluation of Diseases

The degree of illness is evaluated according to the Common Terminology Criteria for Adverse Event v5.0 as follows.

Grade 1: Mild (no need for therapeutic intervention for illness, etc.)

Grade 2: Moderate (Requires therapeutic intervention such as outpatient drug treatment for illness, etc.)

Grade 3: Severe (Requires hospitalization for illness, etc.)

Grade 4: Life-threatening or incapacitated

Grade 5: Death

The causal relationship with protocol treatment is classified into the following five categories

1. Yes: It is clear that the disease has arisen or become severe due to protocol treatment, and it is judged that there is little possibility that it is due to exacerbation of the original disease or other factors (comorbidity, other drugs/treatments, incidental occurrence).
2. Probably: It is unlikely that the disease was caused or aggravated by exacerbation of the original disease or other factors (comorbidities, other drugs and treatments, incidental cases), and it is more plausible to assume that it is due to protocol treatment.
3. Possibility: It is difficult to determine whether the disease was caused or severed by protocol treatment, or whether it was due to exacerbation of the underlying disease or other factors (comorbidity, other drugs or treatments, incidental occurrence).
4. Unlikely: It is unlikely that the disease was caused by protocol treatment, and it is considered plausible to think that it is due to exacerbation of the underlying disease or other factors (comorbidity, other medications and treatments, incidents).
5. None: It is clear that the disease has arisen or become severe due to exacerbation of the original disease or other factors (comorbidities, other drugs or treatments, incidental cases), and it is judged that there is little possibility of protocol treatment.

Among the above classifications, if it is judged that either (1) ~ (3) is determined, it is considered to be "causal", and if it is judged that either (4) or (5) is determined, it is considered "not causal".

A "serious illness" is defined as one that falls under any of the following

(1) Death

(2) Diseases that may lead to death

(3) Diseases that require hospitalization at a medical institution or extension of the period of hospitalization for treatment

(4) Disability

(5) Diseases that may lead to disability

(6) Diseases that are as serious as (3) to (5) and death or diseases that may lead to death, etc.

(7) Congenital diseases or abnormalities in later generations

## 10.3. Foreseeable Diseases

Since enteral nutrition is not a pharmaceutical product, there is no data on adverse events, but gastrointestinal symptoms (diarrhea, nausea and vomiting, abdominal pain, intestinal ischemia, ileus, melena) are expected as in general EN. In the NUTRIREA-2 study, which examined early EN overseas, diarrhea (36%), vomiting (34%), and intestinal ischemia (2) were found in the EN group. %) and ileus (1%).^17)^

Therefore, among these gastrointestinal symptoms, Intestinal ischemia is defined as one of the following: there is no blood flow in any of the major arteries that supply blood to the intestinal tract (superior mesenteric artery, inferior mesenteric artery, or iliac artery), there is evidence of intestinal wall damage on imaging (CT angiography, angiography, or magnetic resonance angiography), there are endoscopic criteria for colorectal ischemia according to the Favier classification system (stage I, Findings of petechiae, stage II, petechiae and superficial ulcers, stage III, necrotic ulcers and polyp-like lesions), and intestinal ischemia during surgery.

## 10.4. Measures to be taken in the event of illness, etc.

10.4.1. Measures for Research Subjects

In the event of an illness, the Principal Investigator or Co-Investigator shall take appropriate measures to ensure the safety of the Research Subject, such as discontinuing treatment and administration of the test product.

If the patient is still ill at the time of the last observation at the end of the test article, the investigator or co-investigator will continue to follow up until the patient recovers to the baseline value (Grade) or until the patient is clinically stabilized.

10.4.2. Evaluation & Recording

The principal investigator or co-investigator shall describe the name of the disease, the date of onset, the severity of the disease, whether it is serious or non-serious, the details of treatment and treatment, and the outcome (when to recover if recovered, and when to recover if symptoms are fixed) in the original materials (medical records, etc.).

10.4.3. Reporting of serious illnesses, etc.

In the event that the investigator of the conducting medical institution becomes aware of the occurrence of a serious disease, the principal investigator of the conducting medical institution shall promptly report the disease to the administrator of the conducting medical institution and notify the principal investigator. The principal investigator shall report the illness and comply with the reporting deadline in accordance with "Table 10.4.3 Deadline for Reporting the Occurrence of Serious Illness, etc."

Table 10.4.3 Reporting Deadlines for Serious Illness

| Diseases, etc. | Predictability | Deadline for Reporting to Managers and Committees | PMDA  Reporting deadline to |
| --- | --- | --- | --- |
| - 1. Death | UnpredictableNote 1) | Within 7 days | Within 7 days |
|  | predictable | Within 15 days | Periodic ReportNote 2) |
| - 1. Diseases that may lead to death | unpredictable | Within 7 days | Within 7 days |
|  | predictable | Within 15 days | Periodic Reporting |
| - 1. Diseases that require hospitalization at a medical institution or extension of the period of hospitalization for treatment | unpredictable | Within 15 days | Within 15 days |
|  | predictable | Periodic Reporting | Periodic Reporting |
| - 1. Obstacle | unpredictable | Within 15 days | Within 15 days |
|  | predictable | Periodic Reporting | Periodic Reporting |
| - 1. Diseases that may lead to disability | unpredictable | Within 15 days | Within 15 days |
|  | predictable | Periodic Reporting | Periodic Reporting |
| - 1. Diseases that are serious in accordance with (1) to (5) above | unpredictable | Within 15 days | Within 15 days |
|  | predictable | Periodic Reporting | Periodic Reporting |
| - 1. Congenital diseases or disorders in later generations | unpredictable | Within 15 days | Within 15 days |
|  | predictable | Periodic Reporting | Periodic Reporting |

Note 1): Definition of "unpredictable" disease, etc.: Refers to a disease that is not described in a document that outlines pharmaceuticals, etc., or that is described but whose nature and severity do not match the description. < If the definition is described in the research protocol, the definition here is not necessary. >

Note 2): The number of reports of diseases, etc., based on Article 13 of the Act is reported in accordance with the report on the implementation status of specific clinical research in Appendix 3 "Periodic Report".

10.4.4. Reporting Illness

In the event that the investigator of the conducting medical institution becomes aware of the occurrence of a disease, the principal investigator of the conducting medical institution shall report it to the administrator of the implementing medical institution when making a periodic report every year. The principal investigator shall report the disease to the Certified Clinical Research Review Board described in the implementation plan. The principal investigator shall promptly provide information to the other investigators, who shall promptly report the contents of the provision of such information to the administrator of the medical institution. Every year, the report of illness shall be made within two months after the expiration of the relevant period, and the reporting deadline shall be complied with.

10.4.5. Bug Reporting

Not applicable

## 10.5. Adverse Events Excluding Illness

10.5.1. Definition of Adverse Events

Adverse events are all undesirable or unintended injuries or illnesses that occur in the study subjects, regardless of whether or not there is a causal relationship, or their signs (including abnormalities in laboratory values), including cases in which an existing disease (not including the underlying disease) worsens during the study period.

10.5.2. Assessment of adverse events

For the evaluation of adverse events, 10.2 shall be applied mutatis mutandis.

10.5.3. Measures for Research Subjects

10.4.1 applies mutatis mutandis to the measures taken for research subjects.

10.5.4. Evaluation and Recording

For evaluation and recording, 10.4.2 shall apply mutatis mutandis.

10.5.5. Reporting of serious adverse events

In the event of a serious adverse event other than illness at each medical institution conducting the multicenter clinical study, The principal investigator of the research site shall promptly report to the principal investigator, who shall notify the investigator of each medical institution conducting the joint research as necessary.

# **11. DATA MANAGEMENT**

In this study, data center personnel implement data management. Outliers and outliers confirmed by data monitoring in the data entered in the electronic case report form are checked and corrected as queries on the electronic case report form. After the data is fixed in the data center, the fixed data is provided to the statistical analysis manager. Details are stipulated in the data management plan.

# **12. Effectiveness and Safety Evaluation Committee**

In this study, no efficacy safety evaluation committee was established.

# **13. Management of compliance, modification and non-conformity with the research plan (deviation from the research plan, etc.)**

## 13.1. Complying with the study plan

The Principal Investigator or Co-Investigator shall not conduct any deviation (i.e., deviation or modification from the Research Plan) that does not conform to the research plan without the prior consent of the principal investigator and the prior written approval of the Accredited Clinical Research Review Board as stated in the implementation plan.

## 13.2. Changes to the Research Proposal

13.2.1. Procedure for Changing the Research Proposal

If you want to change the contents of the research plan, follow the steps below.

1) If the principal investigator deems it necessary to change the research plan, the principal investigator shall provide the principal investigator with a proposed revision of the research plan and other necessary materials and information.

2) The Principal Investigator shall give the Principal Investigator the time necessary for him or her to fully examine the proposed amendment to the research plan and other materials and information provided by the Principal Investigator in accordance with the preceding paragraph, and to discuss with the Principal Investigator.

3) After obtaining the consent of the principal investigator, the principal investigator shall prepare an implementation plan as necessary from the document describing the changes and the revised research plan.

4) The principal investigator shall listen to the opinion of the Accredited Clinical Research Review Committee described in the implementation plan and obtain approval for the revised research plan.

5) Based on the results of the examination, the principal investigator will report to the principal investigator and obtain approval from the administrator of the medical institution.

## 13.3. Management (Non-compliance with research plans, etc.)

1) When the Principal Investigator or Co-Investigator learns that a patient is not compliant, he or she shall promptly report the case to the administrator of the medical institution and notify the principal investigator of the case.

2) In the event that a serious substance is found that affects the human rights, safety, progress of the research, and reliability of the results of the study (e.g., non-compliance with the inclusion/exclusion criteria, discontinuation criteria, prohibition of concomitant therapy, etc.), the principal investigator shall promptly obtain the opinion of the Accredited Clinical Research Review Board described in the implementation plan as a serious non-conformity.

3) Serious non-conformity does not include those who did not comply with the research plan for other medically compelling reasons in order to avoid immediate risk to the subjects of clinical research.

# **14. Ethical Matters**

## 14.1 Rules and Regulations to be complied

This study will be conducted in accordance with the ethical principles based on the Declaration of Helsinki, in accordance with the Clinical Trials Act, the Enforcement Regulations of the Act, the Personal Information Protection Act, and other related notices.

## 14.2. Handling of Personal Information, etc.

In this study, when enrolling patients, The research subject identification code consists of numeric symbols that are unrelated to information that can identify a specific individual, such as initials and medical record IDs, and when preparing documents related to this research, such as case registration slips and case report forms, the research subject identification code is used to prevent individual identification. We will create a table for managing personal information (hereinafter referred to as "correspondence table"), which links information containing information such as the name and medical record ID of the research subject so that the research subject can be identified, and strictly store and manage it so that it will not be leaked to the outside.

This research is a multi-center joint research conducted at multiple facilities including Yokohama City University Hospital, and a table for managing personal information (hereinafter referred to as the "correspondence table") that links samples and information that cannot be used to identify individuals at each medical institution with information such as the name and medical record ID of the research subject so that the research subject can be identified will be appropriately managed. When providing the case registration slip, case report form, test data, etc. of the research subject outside the research institution, the research subject identification code or registration number shall be used (limited to those that have been processed or managed so that it is not immediately possible to determine which research subject's sample or information is．

## 14.3. Expected benefits and disadvantages of research subjects as a result of research participation

14.3.1. Anticipated Profits

Participation in this study does not directly benefit the study subjects, and there is a possibility that participation in the study will contribute to future medical advances.

14.3.2. Anticipated Disadvantages

The test product used in this study has not been approved by the regulatory government regarding its efficacy and effect on the disease to be tested, and is not covered by insurance, but it is generally covered by insurance as a concentrated liquid food. Compared to routine medical care, there is no economic burden for the study subjects to participate in this study. The principal investigator or co-investigator of the study shall be required to refer to "10.4. Measures to be taken in the event of the occurrence of illness, etc."

## 14.4. Handling of research results (including accidental findings) pertaining to research subjects

Although there is no possibility that this study will provide important knowledge about the health of the research subject, genetic characteristics that may be passed on to the descendants, etc., if information (including incidental findings) that has a significant impact on the health of the research subject is obtained through the tests to be performed, the principal investigator or the co-investigator will explain to the research subject, In addition, the results of the study subject's participation in the study will be explained to the study subject himself or herself in the course of medical treatment.

## 14.5. Viewing Sources

In this study, the principal investigator and the conducting medical institution will directly inspect all clinical research-related records, including original documents, during monitoring and auditing related to the clinical study, and during investigations by the Accredited Clinical Research Review Board and regulatory authorities.

# **15. Procedures for Obtaining Informed Consent**

Before enrolling a patient, the Principal Investigator and Co-Investigator shall fully explain the following matters using the latest consent and explanatory documents approved by the Accredited Clinical Research Review Board and approved by the administrator of the site described in the implementation plan, and confirm that the patient fully understands the contents. If the principal investigator at each site deems it necessary, the explanatory documents and consent forms may be changed for each site, but they must be submitted to the principal investigator and approved by the Accredited Clinical Research Review Board as described in the implementation plan. ．

As a general rule, the matters to be explained to the research subjects at the time of obtaining informed consent are as follows, except for matters approved by the administrator of the conducting medical institution after the opinion of the Accredited Clinical Research Review Board.

1) The name of the specific clinical study to be conducted, the fact that the implementation of the specific clinical research has been approved by the administrator of the conducting medical institution, and the fact that the implementation plan has been submitted to the Minister of Health, Labour and Welfare

2) The name of the medical institution and the name and title of the principal investigator (including the name and title of the principal investigator and the name and title of the principal investigator of the conducting medical institution in the case of conducting specific clinical research as a multicenter joint research, as well as the name and title of the principal investigator of the conducting medical institution.)

3) Reasons for being selected as a subject for specific clinical research

4) Anticipated benefits and disadvantages of conducting specific clinical research

5) Refusal to participate in specific clinical research is voluntary.

6) Matters concerning the withdrawal of consent

7) The fact that you will not be treated unfavorably by refusing to participate in specific clinical research or withdrawing your consent

8) Method of Disclosure of Information on Specific Clinical Research

9) The fact that the subject of the specified clinical research or the person who has assigned the subject of the specified clinical research (hereinafter referred to as the "subject of the specified clinical research, etc.") can obtain or view the research plan and other materials related to the implementation of the specified clinical research at the request of the person or the person who has assigned the subject of the specified clinical research (hereinafter referred to as the "subject of the specified clinical research, etc."), and the method of obtaining or viewing the such information

10) Matters concerning the protection of personal information of subjects of specific clinical research

11) Secondary use of samples and information

12) Methods of storage and disposal of samples, etc.

13) Status of involvement in specific clinical research as stipulated in each item of Article 21, Paragraph 1 of the Ordinance for Enforcement of the Clinical Trials Act

14) System for Responding to Complaints and Inquiries

15) Expenses related to the implementation of specific clinical research

16) Comparison of the presence and content of other treatments and the expected benefits and disadvantages of other treatments

17) Compensation for health damage caused by the implementation of specific clinical research and provision of medical care

18) Matters to be examined by the Accredited Clinical Research Review Committee, which conducts review and opinion services for specific clinical research, and other matters related to the Accredited Clinical Research Review Board pertaining to the specific clinical research

19) Other matters necessary for the implementation of specific clinical research

## 15.1. Responding to Consultations from Research Subjects and Their Related Parties

If the method of response is unclear, the investigator or the investigator will respond to the consultation from the research subject, etc., and the research secretariat will respond according to the content of the consultation.

## 15.2. When obtaining informed consent from a substitute, etc.

Since this study was conducted on ICU patients, it may not be possible to obtain written consent directly from the patient. In that case, consent shall be obtained from the parents, siblings, children/grandchildren, grandparents, relatives living together, or those who are considered to be equivalent to their close relatives as a substitute, and a record of the consent shall be kept showing the relationship between the substitute and the patient.

## 15.3. Obtaining Informed Ascent

Not applicable in this study because it included patients of adult age or older.

## 15.4. When you withdraw your consent after giving your consent

If the patient wishes to revoke his or her consent to participate in the research after obtaining his or her consent to participate in the research, the consent shall be withdrawn.

When withdrawing consent or expressing intention to withdraw consent orally or by letter, it should be recorded in the medical record, and efforts should be made to conduct possible discontinuation tests and safety investigations with maximum consideration for the safety of the research subject.

Withdrawal of consent means withdrawal of consent to participate in the study, and is distinguished from refusal to continue protocol treatment ((1) below).

(2) In the case of withdrawal of consent, the follow-up request according to the subsequent protocol is discontinued, and in the case of (3), the patient data of all withdrawal of consent are excluded from the analysis due to the handling of the data.

The procedure for discontinuing the follow-up request for the patient and deleting the patient data shall be separately stipulated in the procedure manual, and the completion of each task shall be reported to the principal investigator.

(1) Patient refusal: refusal to continue protocol treatment thereafter (follow-up continues).

(2) Withdrawal of consent: Withdraw consent to participate in the study and prevent all subsequent treatment and follow-up according to the protocol.

(3) Withdrawal of all consent: Withdraw consent to participate in the study and make all data from the time of participation in the study, including information at the time of registration, not available for use in the study.

## 15.5. When it is not necessary to obtain the consent of the specified clinical research subject, etc.

Not applicable in this study.

# **16. Methods of storage and disposal of samples and information**

[Specimen storage]

The remaining samples and serum that have been measured for clinical testing among the samples collected in this study should be promptly disposed of in accordance with the procedures of laboratories, hospitals, and universities.

Only subjects with consent obtained as an option, plasma on Day 0 (before the start of administration), Day 3 and Day 7, After anonymization at the medical institution where the sample was collected, the specimen is transported to the Department of Anesthesiology, Yokohama City University, according to a separately specified procedure, and stored for 5 years after the end of the study, and used for secondary analysis.

[Storage of Information]

When storing electronic data, set a password, store it on a personal computer or electromagnetic recording medium such as a USB memory that is independent of the hospital LAN or the Internet, and strictly store and manage it in a lockable vault when not in use. The period shall be 5 years from the date of the report on the termination of the study or 3 years from the date of the final publication of the results of the study, whichever is later (however, information that has been processed so that individuals cannot be identified will be kept for an indefinite period even after the end of the retention period due to the possibility of secondary use).

The storage method is managed according to the type of information as follows.

What kind of information do we store?

・Information that includes information that can identify an individual of a research subject (a table for managing personal information that can identify a research subject from the research subject identification code (hereinafter referred to as the "correspondence table"), consent form, consent withdrawal form, etc., for the purpose of identifying and inquiring about the research subject)

・Information that has been processed so that the individual of the research subject cannot be identified.

・Research-related materials (research plans, other examination documents, etc.)

Each is stored as follows.

Documents and records that have passed the retention period shall be disposed of with the utmost care to prevent leakage of personal information and confidential information, paper media shall be shredded and disposed of, and other media shall be disposed of by appropriate methods such as deletion after making it impossible to identify individuals.

1) Matters to identify research subjects (correspondence table)

2) Matters related to medical treatment and examinations for research subjects

3) Matters related to participation in specific clinical research (case registration form)

4) Research Proposal

5) Implementation plan

6) Documents, Consent Forms, and Consent Withdrawal Forms

7) Consent form (original with signature)

8) Case report form (copy)

9) Documents submitted to the Accredited Clinical Research Review Board

10) Notification of the results of the Accredited Clinical Research Review Board and a letter of approval from the administrator of the implementing medical institution

11) Documents related to monitoring and auditing (when conducting audits)

12) Contracts for the Implementation of Specific Clinical Research

(Excluding those related to contracts concluded with pharmaceutical manufacturers and distributors or their special related parties.)

13) Other documents or records related to this research

## 16.1. Secondary Use of Samples and Information

Additional analyses of lipid metabolism and protein metabolism were performed on plasma, whole blood, and feces on stored Day0 (before administration of the intervention preparation) Day3 and Day7 to analyze the effects of diarrhea on nutrient metabolism in severe disease.

In the case of the possibility that the researchers involved in this study may use the information and research data obtained in this study for different research purposes or provide it to other research institutions, in such cases, a new research plan will be prepared and will be implemented after obtaining approval from the Ethical Review Committee, which should listen to the research.

## 16.2. Use of Samples and Information as a Biobank

Not applicable in this study.

# **17. Research on the management of the opposite of interests, etc**

## 17.1. Sources and Financial Relationships

This study will be conducted with the support of Otsuka Pharmaceutical Factory, Ltd., which provides information on the test product, but is not involved in the conduct, analysis, or reporting of the study.

## 17.2. Management of Conflicts of Interest

17.2.1. Management of Conflicts of Interest of the Principal Investigator (or Principal Investigator)

This clinical study was conducted with research funding from Otsuka Pharmaceutical Factory, Inc., and the test product used in the study, Hinex Renut, will be provided free of charge by Otsuka Pharmaceutical Factory Co., Ltd., the manufacturer and distributor of Hanex lute. We have concluded a contract for matters stipulated in the Clinical Research Act and related laws and regulations.The Principal Investigator shall establish Conflict of Interest Management Standards, confirm the facts by the administrator of the Conducting Medical Institution, and prepare a Conflict of Interest Management Plan.

17.2.2. Study, share the interests of physicians, etc

In this study, the principal investigator and the chief statistical analyst who will be the reporting of the conflict of interest will confirm the facts with the administrator of the conducting medical institution and obtain a report confirming the conflict of interest.

# **18.　Expenses and remuneration for research subjects**

Among the costs incurred in conducting this study, the medical expenses required for each patient (examination fee, hospitalization fee, drug cost, examination fee, etc.) will be paid by the study participant because all the treatment performed in this study is covered by insurance. There is no burden on the research subjects because the Hi-Nexus Renut, which is the test product, is provided by Otsuka Pharmaceutical Factory Co., Ltd., and the GLUCERNA® REX is provided by the principal investigator.

# **19. Indemnity for Health Hazards**

In the event of health damage to the research subject due to participation in this study, the principal investigator and the co-investigator will provide appropriate treatment and other necessary measures.

In addition, in preparation for compensation in the event of liability due to health damage caused by this research or in the event of death or permanent disability of the research subject 1 ~ 3 health damage, we will take out clinical research liability insurance.

(1) When there is a significant deviation from the research plan

(2) In the event of intentional or negligent misconduct or medical error on the part of the principal investigator or co-investigator of the study.

(3) In the event of an illegal act or default by a third party

(4) When there is willful misconduct or gross negligence on the part of the research subject, etc.

# **20. Periodic Reporting**

## 20.1. Periodic Reporting to the Accredited Clinical Research Review Board

The principal investigator shall report the status of the implementation of the specified clinical research to the administrator of the implementing medical institution every one year (within two months after the expiration of the period) from the date of submission of the implementation plan to the Minister of Health, Labour and Welfare, and shall make periodic reports to the Accredited Clinical Research Review Board described in the implementation plan.

(1) Number of clinical research participants

(2) Occurrence of diseases and subsequent course

(3) Occurrence of non-conformity and subsequent response

(4) Evaluation of safety and scientific validity

(5) Matters related to the involvement of pharmaceutical manufacturers, distributors, etc., as stipulated in the Conflict of Interest Management Standards

When the Principal Investigator reports to the Accredited Clinical Research Review Board, the Principal Investigator shall promptly provide information to the other Principal Investigators.

## 20.2. Periodic Report to the Minister of Health, Labour and Welfare

(Example sentences)

The Principal Investigator (or Principal Investigator) shall report to the Minister of Health, Labour and Welfare on the following matters within one month from the date on which the Certified Clinical Research Review Committee stated in the Implementation Plan gives an opinion on the implementation status of the Specific Clinical Research.

(1) The name of the Accredited Clinical Research Review Board listed in the implementation plan

(2) Appropriateness of continuation of the specific clinical research by the Accredited Clinical Research Review Board

(3) Number of Specified Clinical Research Subjects Participating in Specified Clinical Research

# **21. Disclosure of Research Information and Results**

## 21.1. Registration of studies

Prior to the implementation of this research, the results of the study will be recorded (registered) in a database (jRCT = Japan Registry of Clinical Trials) maintained by the Ministry of Health, Labour and Welfare.

## 21.2. Publication of Research Results

The principal investigator shall prepare a primary endpoint report or summary report and a summary thereof, and the deadline for preparing the report shall be within one year after the end of the period for collecting data related to the main endpoint or all endpoints.

When the principal investigator prepares the primary endpoint report or summary report and its summary, the Certified Clinical Research Review Committee described in the implementation plan hears the opinions and submits it to the administrator of the implementing institution without delay.Record (register) in the jRCT, "without delay," means within one month from the date on which the committee expresses its opinion. The principal investigator shall provide information to the other investigators at the time of submission to the administrator of the conducting medical institution, and the other principal investigator shall promptly report the content of the provision of the information to the administrator of the other conducting medical institution.

## 21.3. Publication of Academic Societies, etc.

The results obtained from this research will be promptly announced at academic conferences or submitted to papers. When making a public announcement, the results will be made after taking necessary measures to protect the human rights of the research subjects and their related parties, or the rights and interests of the researchers, etc. and their related parties.

# **22. Quality Management and Quality Guarantee**

## 22.1. Monitoring

When conducting monitoring, the principal investigator shall carry out the following matters

1. Efforts are made to protect the human rights and safety of research subjects.
2. Clinical research must be conducted in compliance with the latest implementation plan, research protocol, and ministerial ordinances.
3. Written consent has been obtained from the research subject to conduct the clinical study.
4. Verify the accuracy of records, etc. against original materials, etc.

In this study, central monitoring will be performed based on the CRF data collected in the data center, and on-site monitoring will not be performed to compare the original data with the CRFThe monitoring staff conducts central monitoring twice a year at the request of the principal investigator, and the prepared periodic monitoring report is submitted to the principal investigator for review, and the information on the problems pointed out is shared with the researchers at each research institution and efforts are made to improve it.

## 22.2. Auditing

In this study, no audits were conducted.

# **23. Attribution of Research Results (Intellectual Property Rights)**

In the event that an intellectual property right arises as a result of this research, the right belongs to Yokohama City University.

# **24. Research Implementation Structure**

## 24.1. Principal Investigator (or Principal Investigator)

Name of research institution: Yokohama City University Hospital, Department of Intensive Care

Research institution address: 3-9 Fukuura, Kanazawa-ku, Yokohama-shi, Kanagawa

Research InstituteTEL: 045-787-2918

Principal Investigator: Kensuke Nakamura

Principal Investigator: Intensive Care Unit

Role: Supervision and execution of research

## 24.2. Research Secretariat

Name: Nobuyuki Yokoyama

Affiliation: Yokohama City University Hospital, Intensive Care Department

Address: 〒236-0004 Kanagawa, Yokohama, Kanazawa-ku, Fukuura 3-9

TEL：045-787-2918

Role: Conducting research, liaison and coordination with joint research institutes

## 24.3. Investigator and Investigator

Table 24.3 List of medical institutions and principal investigators

Role: Patient Enrollment, Implementation of Protocol Treatment

| Implementing Medical Institutions | affiliation | Title | Principal investigator |
| --- | --- | --- | --- |
| Yokohama City University Hospital  〒236-0004 Kanagawa, Yokohama, Kanazawa-ku, Fukuura 3-9  045-787-2918 | Intensive Care Unit | doctor | Kensuke Nakamura |
| Keio University Hospital  〒160-0016 35 Shinanomachi, Shinjuku-ku, Tokyo  03-3353-1211 | Emergency Department | doctor | Junichi Sasaki |
| St. Marianna College of Medicine  〒216-8511 Kanagawa Prefecture Kawasaki City Miyamae Ward Sugo 2-16-1  044-977-8111 | Emergency Medicine | doctor | Akihito Nagatomi |
| St. Marianna University School of Medicine Yokohama City Seibu Hospital  〒241-0811 Kanagawa-ken, Yokohama-shi, Asahi-ku, Yashi-cho 1197-1  045-366-1111 | Critical Care Center | doctor | Minoru Yoshida |
| Hitachi General Hospital  〒317-0077 2-1-1, Jonan-cho, Hitachi City, Ibaraki  0294-23-1111 | Department of Emergency and Intensive Care | doctor | Yuji Takahashi |
| Osaka Medical and Pharmaceutical University  〒569-8686 2-7 Gakumachi, Takatsuki City, Osaka Prefecture  072-683-1221 | Department of Emergency Medicine | doctor | Junji Hatakeyama |

## 24.4. Data Management Officer

Name: Shizuka Kashiwagi

Affiliation: Yokohama City University Hospital, Intensive Care Department

Address: 〒236-0004 Kanagawa, Yokohama, Kanazawa-ku, Fukuura 3-9

TEL：045-787-2918

## 24.5. Statistical Analyst

Satoru Shinoda

Affiliation: Department of Clinical Statistics, Yokohama City University School of Medicine

Location: 〒236-0004 3-9 Fukuura, Kanazawa-ku, Yokohama-shi, Kanagawa

Phone number: 045-787-2572

## 24.6. Who is responsible for monitoring?

Name: Shizuka Kashiwagi

Affiliation: Yokohama City University Hospital, Intensive Care Department

Address: 〒236-0004 Kanagawa, Yokohama, Kanazawa-ku, Fukuura 3-9

TEL：045-787-2918

## 24.7. Who is responsible for the audit?

Not applicable

## 24.8. R&D Planning Officer

Not applicable

## 24.9. Coordination Management Practitioners

Not applicable

## 24.10. Persons other than the principal investigator who supervise the research

Not applicable

## 24.11. Other clinical laboratories and medical and technical departments and institutions related to clinical research.

Not applicable

## 24.12. Outsourcing Organizations

The construction of an electronic database, randomization assignment, and account issuance related to this research will be outsourced to TXP Medical Co., Ltd., and the implementation status of the outsourced work will be supervised based on the consignment agreement.

Institution name: TXP Medical Co., Ltd.

Address: 〒101-0042 Tokyo, Chiyoda-ku, Kanda Higashimatsushita-cho 41-1 H¹O Kanda 706

Details of outsourced work: Electronic database construction, randomization allocation, account issuance

Person in charge: Tomohiro Sono

# **25. References**

1) Reintam Blaser A, Starkopf J, Alhazzani W, et al. (2017) Early enteral nutrition in critically ill patients: ESCIM clinical practice guidelines. Intensive Care Med ; 43:380e98

2) Singer, P., Reintam, A., Berger, M. M., et al. (2019) ESPEN Guideline ESPEN guideline on clinical nutrition in the intensive care unit. Clinical Nutrition, 38(1), 48–79

3) McClave, S. A., Taylor, B. E., Martindale, R. G., et al. (2016). Guidelines for the Provision and Assessment of Nutrition Support Therapy in the Adult Critically Ill Patient: Society of Critical Care Medicine (SCCM) and American Society for Parenteral and Enteral Nutrition (A.S.P.E.N.). Journal of Parenteral and Enteral Nutrition, 40(2), 159–211

4) Reintam A, Parm P, Kitus R, et al. (2009) Gastrointestinal symptoms in intensive care patients. Acta Anaesthesiol Scand.; 53:318-24

5) McClave SA, Sexton LK, Spain DA, et al. (1999) Enteral tube feeding in the intensive care unit: factors impeding adequate delivery. Crit Care Med; 27: 1252–1256.

6) Montejo JC. (1999) Enteral nutrition-related gastrointestinal complications in critically ill patients: a multicenter study. The Nutritional and Metabolic Working Group of the Spanish Society of Intensive Care Medicine and Coronary Units. Crit Care Med; 27: 1447–1453.

7) Reintam Blaser A, Poeze M, Malbrain M, et al. (2013) Gastrointestinal symptoms during the first week of intensive care are associated with poor outcome: a prospective multicentre study. Intensive Care Med; 39: 899–909.

8) McRorie, J. W., & McKeown, N. M. (2017) Understanding the Physics of Functional Fibers in the Gastrointestinal Tract: An Evidence-Based Approach to Resolving Enduring Misconceptions about Insoluble and Soluble Fiber. Journal of the Academy of Nutrition and Dietetics, 117(2), 251–264

9） Kazuo Hino, Sho Miyatake, Fumio Yamada, Naoyuki Endo, Ryosuke Akiyama, Goro Ebisu . Undigested low-methoxy pectin prevents diarrhea and induces colonic contraction during liquid-diet feeding in rats. Nutrition 78 (2020) 110804

10) Nakamura, K., Inokuchi, R., Fukushima, K., et al. (2019) Pectin-containing liquid enteral nutrition for critical care: a historical control and propensity score matched study. Asia Pacific Journal of Clinical Nutrition, 28(1), 57–63

11) Dionne, J. C., Mbuagbaw, L. (2023). Diarrhea in the critically ill: definitions, epidemiology, risk factors and outcomes. In Current Opinion in Critical Care, Vol. 29, Issue 2, pp. 138–144

12)Lewis, S. J., & Heaton, K. W. (1997). Stool form scale as a useful guide to intestinal transit time. Scandinavian Journal of Gastroenterology, 32(9), 920–924.

13) Vincent JL, Moreno R, Takala J, et al. (1996). The SOFA (Sepsis-related Organ Failure Assessment) score to describe organ dysfunction/failure. On behalf of the Working Group on Sepsis-Related Problems of the European Society of Intensive Care Medicine. Intensive Care Med. Jul; 22(7):707-10. doi: 10.1007/BF01709751.

.

15) Brisard, L., Le Gouge, A., Lascarrou, J. B., et al. (2014). Impact of early enteral versus parenteral nutrition on mortality in patients requiring mechanical ventilation and catecholamines: Study protocol for a randomized controlled trial (NUTRIREA-2). Trials, 15(1).

16) F abregas N, Ewig S, Torres A, et al. (1999). Clinical diagnosis of ventilator associated pneumonia revisited: comparativevalidation using immediate post-mortem lung biopsies. Thorax ; 54(10):867e73

17) Reignier, J., Boisramé-Helms, J., Brisard, L., Lascarrou, et al. (2018). Enteral versus parenteral early nutrition in ventilated adults with shock: a randomised, controlled, multicentre, open-label, parallel-group study (NUTRIREA-2). The Lancet, 391(10116), 133–143

E1) Taito S, Kawai Y, Liu K,etal. (2019) Diarrhea and patient outcomes in the intensivecare unit: Systematic review and meta-analysis. J Crit Care; 53:142–148

E2）Dionne, J. C., & Mbuagbaw, L. (2023). Diarrhea in the critically ill: definitions, epidemiology, risk factors and outcomes. Current Opinion in Critical Care (Vol. 29, Issue 2, pp. 138–144)

E3）Dionne JC, Mbuagbaw L, Devlin JW,etal. (2022) Diarrhea during critical illness: amulticenter cohort study. Intensive Care Med; 48:570–579.

E4) Dionne JC, Campbell T, Janisse N,etal . (2020) Mo1960 effect of fiber, osmolarity,and protein content of enteral nutrition on the development of diarrhea incritical illness. Gastroenterology; 158:S–994.

E5) Jordan, E. A., Moore, S. C. (2020). Enteral nutrition in critically ill adults: Literature review of protocols. In Nursing in Critical Care (Vol. 25, Issue 1, pp. 24–30).

# **26. Appendix**

Not applicable
