# Supplementary material for: Low-methoxy pectin-containing enteral nutrition in critical care for intestinal tolerance (LOME-PECT): Study protocol for a randomized controlled trial
Source: PLoS One. 2025 Jul 11;20(7):e0326582. doi: 10.1371/journal.pone.0326582 (PMC12250234; doi:10.1371/journal.pone.0326582)
Supplement: S3 File — (DOCX) [file pone.0326582.s004.docx]

研究計画書

研究課題名：

重症患者におけるペクチン含有経腸栄養製剤の腸管不耐

に対する有効性の検証：多施設非盲検化無作為化比較試験

LOME-PECT trial；Low-Methoxy Pectin containing Enteral nutrition in Critically care for intestinal Tolerance

**当該研究を特定する番号**

jRCTs031230684

研究代表医師：　横浜市立大学附属病院　集中治療部　中村 謙介

版数：第2.0版

作成年月日：2024年6月18日

改訂履歴

| 作成日 | 版数 |
| --- | --- |
| 2024年1月15日 | 初版 |
| 2024年6月18日 | 2.0版 |
|  |  |
|  |  |

目次

[**0. 概要** 6](#_Toc169695667)

[0.1. シェーマ 6](#_Toc169695668)

[0.2. 研究の目的と主要評価項目 6](#_Toc169695669)

[0.3. 研究対象者 6](#_Toc169695670)

[0.4. 治療 6](#_Toc169695671)

[0.5. 目標症例数 6](#_Toc169695672)

[0.6. 研究期間 6](#_Toc169695673)

[0.7.　研究代表医師と問い合わせ先 7](#_Toc169695674)

[**1. 本研究の目的** 8](#_Toc169695675)

[**2. 背景と試験計画の科学的根拠** 8](#_Toc169695676)

[2.1. 対象疾患について 8](#_Toc169695677)

[2.2. 標準治療について 9](#_Toc169695678)

[2.3. 試験治療について 9](#_Toc169695679)

[2.4. 試験デザインと主要評価項目について 9](#_Toc169695680)

[2.5. 本研究の意義 9](#_Toc169695681)

[**3. 試験品等情報** 11](#_Toc169695682)

[3.1. 被験薬等 11](#_Toc169695683)

[3.2. 対照薬等 11](#_Toc169695684)

[3.3. 試験品等の管理 11](#_Toc169695685)

[3.4. 試験品等の品質の確保 12](#_Toc169695686)

[**4. 本研究で用いる規準・定義** 13](#_Toc169695687)

[**5. 研究対象者の選定方針** 14](#_Toc169695688)

[5.1. 選択基準 14](#_Toc169695689)

[5.2. 除外基準 14](#_Toc169695690)

[**6. 研究計画** 15](#_Toc169695691)

[6.1. 研究デザイン 15](#_Toc169695692)

[6.2. 目標症例数 15](#_Toc169695693)

[6.3. 研究期間 15](#_Toc169695694)

[6.4. 施設登録及び症例登録・割付方法 15](#_Toc169695695)

[6.5. 治療計画 16](#_Toc169695696)

[6.6. 研究全体の中止 17](#_Toc169695697)

[**7. 観察・検査・調査・評価項目** 19](#_Toc169695698)

[7.1. スケジュール表 19](#_Toc169695699)

[7.2. 実施スケジュール時期と評価項目 19](#_Toc169695700)

[**8. 評価項目** 22](#_Toc169695701)

[8.1. 主要評価項目 22](#_Toc169695702)

[8.2. 副次評価項目 22](#_Toc169695703)

[8.3. 安全性評価項目 23](#_Toc169695704)

[9. 統計解析 23](#_Toc169695705)

[9.1. 解析対象集団 23](#_Toc169695706)

[9.2. 目標症例数の設定根拠 23](#_Toc169695707)

[**9.3. 統計解析方法** 23](#_Toc169695708)

[**9.4. 中間解析** 24](#_Toc169695709)

[**9.5. 一般的事項** 24](#_Toc169695710)

[**10. 疾病等の取り扱いについて** 25](#_Toc169695711)

[10.1. 疾病等の定義 25](#_Toc169695712)

[10.2. 疾病等の評価 25](#_Toc169695713)

[10.3. 予測できる疾病等 26](#_Toc169695714)

[10.4. 疾病等が発現した場合の措置 26](#_Toc169695715)

[10.5. 疾病等を除く有害事象について 27](#_Toc169695716)

[**11. データマネージメント** 28](#_Toc169695717)

[**12. 効果安全性評価委員会** 28](#_Toc169695718)

[**13. 研究計画書の遵守，変更及び不適合の管理（研究計画書からの逸脱等）について** 29](#_Toc169695719)

[13.1. 研究計画書の遵守 29](#_Toc169695720)

[13.2. 研究計画書の変更 29](#_Toc169695721)

[13.3. 不適合の管理（研究計画書不遵守等） 29](#_Toc169695722)

[**14. 倫理的事項** 30](#_Toc169695723)

[14.1 遵守すべき諸規則 30](#_Toc169695724)

[14.2. 個人情報等の取扱い 30](#_Toc169695725)

[14.3. 研究参加に伴い研究対象者に予期される利益及び不利益等 30](#_Toc169695726)

[14.4. 研究対象者に係る研究結果（偶発的所見を含む．）の取扱い 30](#_Toc169695727)

[14.5. 原資料等の閲覧 31](#_Toc169695728)

[**15. インフォームド・コンセントを受ける手続** 32](#_Toc169695729)

[15.1. 研究対象者等及びその関係者からの相談等への対応 32](#_Toc169695730)

[15.2. 代諾者等からインフォームド・コンセントを受ける場合 33](#_Toc169695731)

[15.3. インフォームド・アセントを得る場合 33](#_Toc169695732)

[15.4. 同意後に同意撤回を受ける場合 33](#_Toc169695733)

[15.5. 特定臨床研究対象者等に対する同意取得が不要な場合 33](#_Toc169695734)

[**16. 試料・情報の保管及び廃棄の方法** 34](#_Toc169695735)

[16.1. 試料・情報の二次利用について 35](#_Toc169695736)

[16.2. 試料・情報のバイオバンクとしての利用 35](#_Toc169695737)

[**17. 研究の資金源等，研究に係る利益相反管理** 35](#_Toc169695738)

[17.1. 資金源及び財政上の関係 35](#_Toc169695739)

[17.2. 利益相反管理 35](#_Toc169695740)

[**18.　研究対象者の費用負担・謝礼について** 36](#_Toc169695741)

[**19. 健康被害に対する補償** 36](#_Toc169695742)

[**20. 定期報告** 36](#_Toc169695743)

[20.1. 認定臨床研究審査委員会への定期報告 36](#_Toc169695744)

[20.2. 厚生労働大臣への定期報告 36](#_Toc169695745)

[**21. 研究の情報公開及び結果公表** 37](#_Toc169695746)

[21.1. 研究の登録 37](#_Toc169695747)

[21.2. 研究結果の公表 37](#_Toc169695748)

[21.3. 学会等の公表 37](#_Toc169695749)

[**22. 品質管理及び品質保証** 38](#_Toc169695750)

[22.1. モニタリング 38](#_Toc169695751)

[22.2. 監査 38](#_Toc169695752)

[**23．研究成果の帰属（知的財産権）** 38](#_Toc169695753)

[**24. 研究の実施体制** 39](#_Toc169695754)

[24.1. 研究責任医師（又は研究代表医師） 39](#_Toc169695755)

[24.2. 研究事務局 39](#_Toc169695756)

[24.3. 実施医療機関及び研究責任医師 39](#_Toc169695757)

[24.4. データマネージメント責任者 40](#_Toc169695758)

[24.5. 統計解析責任者 40](#_Toc169695759)

[24.6. モニタリングに関する責任者 40](#_Toc169695760)

[24.7. 監査に関する責任者 40](#_Toc169695761)

[24.8. 研究・開発計画支援担当者 40](#_Toc169695762)

[24.9. 調整管理実務担当者 40](#_Toc169695763)

[24.10.研究代表医師以外に研究を総括する者 40](#_Toc169695764)

[24.11. その他臨床研究に関連する臨床検査施設並びに医学的及び技術的部門・機関 40](#_Toc169695765)

[24.12. 外部委託機関 40](#_Toc169695766)

[**25. 文献** 41](#_Toc169695767)

[**26. 付録** 42](#_Toc169695768)

**略語表**

| 略語 | 正式名称 | 説明 |
| --- | --- | --- |
| A-aDO_2_ | Alveolar-arterial Oxygen Difference | 肺胞気動脈血酸素分圧較差 |
| BSC | Best Supportive care | 症状緩和のための治療 |
| Ca | Calcium | カルシウム |
| Cre | Creatinine | クレアチニン |
| CRP | C Reactive Protein | C反応タンパク |
| DHA | Docosahexaenoic Acid | ドコサヘキサエン酸 |
| DNR | Do Not Resuscitaion | 蘇生処置拒否 |
| EDC | Electronic Data Capture | 電子データ収集システム |
| EN | Enteral Nutrition | 経腸栄養 |
| EPA | Eicosapentaenoic Acid | エイコサペンタエン酸 |
| FiO_2_ | Fraction of inspiratory Oxygen | 吸入酸素濃度 |
| G-CSF | Granulocyte-Colony Stimulating Factor | 顆粒球コロニー形成刺激因子 |
| GCS | Glasgow Coma Scale | グラスゴー・コーマ・スケール |
| GRV | Gastric Residual Volume | 胃内残存量 |
| Hct | Hematocrit | ヘマトクリット |
| HDL | High Density Lipoprotein | 高比重リポタンパク |
| ICU | Intensive Care Unit | 集中治療室 |
| jRCT | Japan Registry of Clinical Trials | 臨床研究等提出・公開システム |
| K | Kalium | カリウム |
| LDL | Low Density Lipoprotein | 低比重リポタンパク |
| Mg | Magnesium | マグネシウム |
| Na | Natrium | ナトリウム |
| PaO_2_ | Partial pressure of arterial Oxygen | 動脈血酸素分圧 |
| PEG | Percutaneous Endoscopic Gastrostomy | （経皮的に増設された）胃ろう |
| PTEG | Percutaneous Trans-Esophageal Gastro-tubing | （経皮的に頚部食道より挿入された）胃管 |
| RCT | Randomized Control Trial | 無作為化比較研究 |
| SOFA | Sequential Organ Fairure Assessment | 多臓器不全の評価スコア |
| TG | TolyGlyceride | 中性脂肪 |
| V | Vitamin | ビタミン |
| WBC | White Blood Cell | 白血球 |

# **0. 概要**

## 0.1. シェーマ

ランダム化比較試験

18歳以上の経腸栄養を行うICU入室患者

説明

同意取得

登録，ランダム割付

試験治療群

ハイネックスリニュートによる経腸栄養

： 3日間投与

最低3日間投与

対照群

グルセルナ-REXによる経腸栄養

： 3日間投与

主要評価項目：

試験品投与開始3日以内の下痢の発生率（Bristol Scale 5-7）

## 0.2. 研究の目的と主要評価項目

目的：

集中治療室（ICU）入室患者で経腸栄養(Enteral nutrition ;EN)を行う者に対し，ペクチン含有のEN製剤ハイネックスリニュートによる腸管不耐（下痢）の減少効果を，ペクチン非含有でエネルギー密度や三大栄養素バランスが同様のEN製剤グルセルナ-REXを対照として検証する．

主要評価項目：

試験品投与開始3日以内の下痢の発生率（Bristol Scale5－7として評価）

## 0.3. 研究対象者

EN製剤でコントロールが必要なICU入室（重症）患者

## 0.4. 治療

対照群：グルセルナ-REXによるEN（持続経胃投与）

試験治療群：ハイネックスリニュートによるEN（持続経胃投与）

## 0.5. 目標症例数

対照群：100例

試験治療群：100例

## 0.6. 研究期間

研究期間：jRCT初回公表日（厚生労働大臣届日）から2028年3月31日

登録期間：jRCT初回公表日（厚生労働大臣届日）から2025年3月31日

臨床研究の対象者の参加予定期間：参加期間は,同意取得日から最長5週間（スクリーニング期間最長1日間，投与開始日～最終観察日まで最長35日間，うち投与期間最大7日間）

## 0.7.　研究代表医師と問い合わせ先

所属：横浜市立大学附属病院　集中治療部

氏名：中村 謙介

問い合わせ先：045-787-2918

# **1. 本研究の目的**

集中治療室（ICU）入室患者で経腸栄養(Enteral nutrition ;EN)を行う者に対し，ペクチン含有のEN製剤ハイネックスリニュートによる腸管不耐（下痢）の減少効果を，ペクチン非含有でエネルギー密度や三大栄養素バランスが同様のEN製剤グルセルナ-REXを対照として検証する．

# **2. 背景と試験計画の科学的根拠**

重症患者（ICU入室患者）において経消化管的に十分な栄養を投与するENの有用性が示唆されており，とくに24～48時間以内のEN開始が感染症発生率を低下させるエビデンスがある^1)^．また，このような重症患者は，高炎症状態によるタンパク質喪失の懸念から，必要十分な栄養投与量の確保が重要である．これに基づき，国際的なガイドラインにおいても，早期からのEN開始が推奨されている^2)3)^．しかし，しばしば合併症によりEN継続が困難となりうる^4）^．とくに下痢は，重症患者の15～50%に起こり，EN減量・中止の原因として多くを占める．また下痢時にはENの吸収が著しく障害される^5)-7)^ため，投与したENが無効ともなりうる．さらに下痢の発生とICU死亡率の悪化やICU滞在期間・入院期間の延長との関連が示されている^E1)^．よってその制御はEN管理上で重要な課題である．

重症病態下での下痢は様々な要因で起こる^E2)^が，複数の先行研究においてEN製剤の選択によっても強い影響を受けることが明らかとなっており^E3,4)^，下痢をしにくいEN製剤を適切に選択する必要がある^5)-7)^．

そこで下痢をしにくいEN整剤として，その成分の食物繊維が注目されている．食物繊維は消化を受けにくい物質群であり，大腸に到達した際に便の水分含有量の適正化に寄与するため，水溶性食物繊維を含有するEN製剤によって，下痢抑制効果が期待される^8)^．中でも水溶性食物繊維の一種であるペクチンは，胃内の酸性環境によってゲル化する性質をもち小腸以降への製剤到達速度を抑制，また小分子ペクチンが特に便の性状を整えて下痢を抑制する効果を持つことが，ラットに小分子ペクチンを投与した際に下痢発生が少ない結果であった基礎研究において明らかにされている^9）^．実際に我々は先行する臨床研究において，低分子ペクチン含有EN製剤による下痢抑制効果を確認している^10)^．

ここで試験製剤であるハイネックスリニュート^Ⓡ^は上述の小分子ペクチンを含むEN製剤であり，同時に50％の脂質と24％のタンパク質を含み高い栄養密度を達成している．しかし栄養濃度や浸透圧，脂質含量が増えれば下痢をきたしやすいため^5)-7)^，本製剤の下痢抑制効果がどの程度あるかは臨床試験で明らかとはされていない．そこで本研究は試験製剤ハイネックスリニュートに対して栄養密度，3大栄養素バランスがもっとも近いペクチン非含有のEN製剤グルセルナ-REXを対照としてENを行い，RCTにおいてペクチン含有EN製剤の下痢抑制効果を検証するものである．

## 2.1. 対象疾患について

EN製剤でコントロールが必要なICU入室（重症）患者

【設定根拠】

ICU入室を要するような重症患者は鎮静・意識障害，呼吸・循環不全のため経口摂取不可であることも多く，経静脈または経消化管な人工栄養の適応となるが，血糖コントロールや感染予防の観点から，より生理的なENの方が望ましいと考えられている．

しかしながら，同時に消化管機能の異常をきたしており，下痢を含むENの副作用により十分な投与が困難となるため，重症患者のEN時の下痢の制御は重要な課題である．

## 2.2. 標準治療について

2.2.1. これまでに実施されてきた標準治療の経緯等

EN製剤は多様な種類があり，具体的にガイドライン等では規定されていなく，その選択には絶対的な規定はなく，患者の病態，施設・部署の慣習，あるいは採用製剤の種類によって総合的に決定される場合が多い．国際的な栄養ガイドラインにおいても，重症病態において糖質と脂質の割合は患者に大きな影響を与えず柔軟に選択できることが謳われている^2)^．

2.2.2. 現在の標準治療

前述のようにEN製剤の選択には絶対的な規定はないが，「グルセルナ^Ⓡ^-REX」は試験製剤に栄養密度・バランスが近く，かつ標準的な組成であり，一般に用いられる製剤の一つである．本製剤をはじめとするペクチン非含有の製剤においては，しばしば下痢の発生を認める．

## 2.3. 試験治療について

ペクチンは植物の葉・茎・果実に含まれる複合多糖類で，消化酵素では分解されない食物繊維であり，一部の市販食品にも添加されている．特に低分子ペクチンは下痢抑制効果に優れることが基礎研究で明らかとなっており，我々の先行研究においても低分子ペクチン含有EN製剤の下痢抑制効果を後ろ向きに検討している．本研究の介入群に用いる「ハイネッックスリニュート」は低分子ペクチンを含んでおり下痢の減少が期待され， それに伴い感染合併症や良好な退院転帰に関連する可能性が期待される．

なお，ペクチンは，果物や野菜に含まれる天然の増粘安定剤であり，ジャムやゼリーなどの食品に広く使用されている．消化器官で分解されずに体内を通過するため，副作用や有害な影響を与えることはないと思われる．欧州食品安全機関（EFSA）やアメリカ食品医薬品局（FDA）などの機関による評価でも，ペクチンは安全であるとされており，日本でも厚生労働省によって安全性が認められている．

ペクチンは，医薬品や化粧品などの分野でも利用されており，投与に伴う医学的なリスクは小さいと推定される．

## 2.4. 試験デザインと主要評価項目について

研究デザイン：多施設ランダム化比較研究

主要評価項目：試験品投与開始3日以内の下痢発生率（Bristol Scale 5-7）

【設定根拠】

EN阻害要因である下痢の抑制は臨床的に重要な課題であり，低分子ペクチン含有EN製剤について，前向きに効果を評価する

下痢の評価法としては， Bristol Scaleを用いる^11)^．人間の糞便の形態を7つのカテゴリーに分類するために設計された指標で,腸のさまざまな疾患の治療効果を評価するための研究ツールとして使われる他,臨床評価幅広く使用されている.

## 2.5. 本研究の意義

重症患者において低分子ペクチン含有EN製剤下痢抑制に与える効果が明らかになることで，特に下痢のリスクが高い場合や，すでにコントロール困難な下痢を来たしている場合のEN製剤選択時に低分子ペクチン含有EN製剤を候補として検討できるようになる．またそのようなEN製剤が存在することを示すことで，集中治療において同様のコンセプトでEN製剤選択をすることで下痢を回避してENを継続する考え方を提示し．ガイドラインを含めて下痢抑制アプローチを提示することができる．特に今回検証する低分子ペクチンをはじめとする食物繊維を集中治療で投与する目的の1つを明確にすることができる．

# **3. 試験品等情報**

## 3.1. 被験薬等

商品名：ハイネックスリニュート^Ⓡ^

試験品名： ハイネックスリニュート

原材料：デキストリン，豚コラーゲンペプチド（ゼラチンを含む），中鎖脂肪酸トリグリセライド，植物油，大豆タンパク酵素分解物（大豆を含む），酵母，藻類由来DHA・EPA含有油，カルニチン，コンブエキス／増粘多糖類，リシン，塩化Mg，ピロリン酸Na，ロイシン，V.C，水酸化K，酸味料，リン酸Ca，イソロイシン，バリン，メチオニン，結晶セルロース，ヒスチジン，フェニルアラニン，ピロリン酸K，トレオニン，トリプトファン，V.B1，香料，V.E，ナイアシン，ピロリン酸鉄，パントテン酸Ca，V.B6，V.B2，V.A，葉酸，V.K2，V.D，V.B12

保管方法：暗所に保存．常温保存はできるが，なるべく冷所に保存．凍結するような場所は避ける．

製造販売会社名：株式会社大塚製薬工場

効能・効果：該当なし

用法・用量（使用方法）：濃厚流動食品（経腸栄養チューブより，持続投与または一日数回ボーラス投与）

剤型（外観）：バッグタイプ（液状）

カロリー濃度1kcal/mL 浸透圧約380mOsm/L タンパク質6.0g/100kcal エネルギー比率　タンパク質24％，炭水化物26％，脂質50％

食物繊維 1.2g/100kcal うちペクチン 0.68g/100kcal配合

## 3.2. 対照薬等

商品名：グルセルナ-REX^Ⓡ^

試験品名：グルセルナ-REX

原材料：なたね油，高オレイン酸ひまわり油，難消化性デキストリン，デキストリン，イソマルツロース，果糖，分離大豆たんぱく，フラクトオリゴ糖，カゼイン，Ca，燕麦線維，塩化Na，ブドウ糖，L-カルニチン，中鎖脂肪酸油/カゼイン，Na，グリセリン，食物レシチン，クエン酸Na，塩化K，myo-イノシトール，クエン酸Ca，炭酸Ca，リン酸Mg，塩化Mg，リン酸K，カラギーナン，ジェランガム，V.C，クエン酸K，グルコン酸亜鉛，硫化鉄，V.E，ナイアシン，グルコン酸銅，パントテン酸Ca，V.A，V.B6，V.B1，V.B2，葉酸，ビオチン，V.D，V.B12

保管方法：直射日光を避け，室温保存．

製造販売会社名：アボットジャパン合同会社

効能・効果：該当なし

用法・用量（使用方法）：濃厚流動食品

剤型（外観）：バッグタイプ（液状）

カロリー濃度1kcal/mL 浸透圧約560mOsm/L タンパク質4.2g/100kcal エネルギー比率　タンパク質17％，炭水化物33％，脂質50％

食物繊維 0.9g/100kcal

## 3.3. 試験品等の管理

研究代表医師は，「試験品管理手順書」を各研究責任医師に配布する．研究責任医師は，所属する実施医療機関の試験品管理担当者に「試験品管理手順書」を渡し，試験品管理担当者は，手順書に従って試験品を適切に管理する．

## 3.4. 試験品等の品質の確保

別途定める「試験品管理手順書」に従う．臨床研究に用いる試験品等の品質が不良である等の情報を得たときには，研究代表医師はその検証を行い，臨床研究の停止等の講ずる措置について，認定臨床研究審査委員会に報告する．また，その記録を作成する．また，研究代表医師は，臨床研究に用いる試験品等の品質が不良である等の理由により，試験品等の回収が必要と判断したときは，速やかに認定臨床研究審査委員会に報告するとともに，以下の業務を行う．研究継続の可否については認定臨床研究審査委員会の審議結果に基づき研究代表医師が判断し，各実施医療機関の責任医師に通知する．

1. 研究分担医師等に対し，試験品等の使用中止と回収の指示を速やかに行う．
2. 回収の内容，原因究明の結果及び改善措置を記載した回収処理記録を作成し，保存する．

# **4. 本研究で用いる規準・定義**

下痢：Bristol scale^12)^

タイプ1 木の実のようなコロコロした硬い固まりの便（兎糞便）

タイプ2 短いソーセージのような固まりの便（塊便）

タイプ3 表面にひび割れのあるソーセージのような便（やや硬い）

タイプ4 表面がなめらかで柔らかいソーセージ,あるいは蛇のようなとぐろを巻く便（普通便）

タイプ5 はっきりとした境界のある柔らかい半分固形の便（軟便）

タイプ6 境界がほぐれてふわふわと柔らかいお粥のような便（泥状便）

タイプ7 固まりのない水のような便（水様便）

SOFA score^13)^

SOFAscore=呼吸系ポイント+心血管系ポイント+腎臓系ポイント+肝臓系ポイント+凝固系ポイント+中枢神経系ポイント


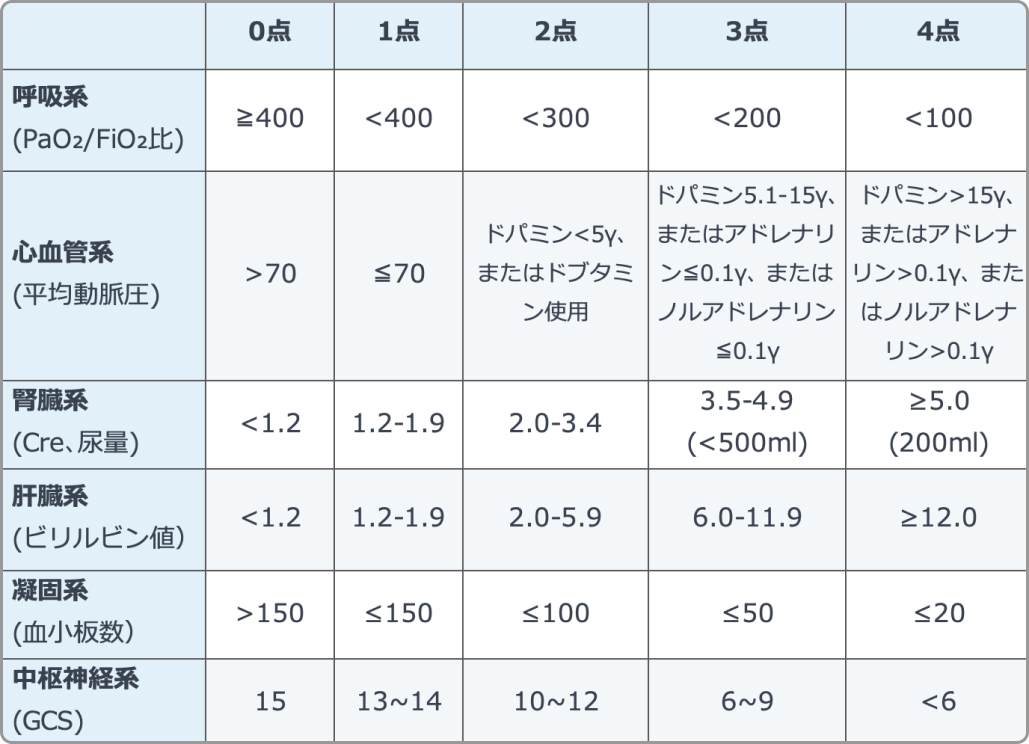


# **5. 研究対象者の選定方針**

## 5.1. 選択基準

(1)ICU入室患者（入室理由は問わない）

(2)登録時年齢が18歳以上の患者

(3)経胃投与によるENが適応となる患者

(4)本研究の参加に関して本人または代諾者による自由意思による同意が文書で得られている患者

【設定根拠】

(1）本研究の重症患者に対する効果を調査するため設定した

(2)本研究は法上の成人年齢を対象とするため設定した

(3) EN投与経路には経胃と経小腸があるが，ペクチンが胃内でゲル化することによる下痢抑制を見込むため，経胃投与のみを対象とする

(4)研究対象者の人権保護と,安全性確保のため設定した

## 5.2. 除外基準

(1)登録前30日以内にENが実施されていた患者

(2)登録時に下痢を認める患者；Bristol scale≧5（Bristol scale 5，6，7点）

(3)本栄養剤の経胃投与によるENが禁忌または医学的に不適切（アレルギーを含む）な患者

(4)DNR/BSCの方針の患者

(5)PEG/PTEGによってENが実施される患者

(6)その他，医師の判断により不適切と判断された場合

【設定根拠】

(1)直近のENが行われた場合，他製剤の影響が否定できないため設定した

(2)すでに下痢を来たしている場合，本製剤による下痢と区別困難であるため設定した

(3)研究対象者の安全性確保のため，また正確な安全性・有効性評価のため設定した

(4)DNR/BSC患者は，苦痛緩和が最優先され，通常とは異なる栄養投与量・方法となる場合が多いため設定した

(5)PEG/PTEGからの投与は例外的であり，標準的な経鼻栄養チューブからの投与とは物理的動態が異なる可能性があるため設定した

(6)本研究に適切な研究対象者を組み入れるため設定した

# **6. 研究計画**

## 6.1. 研究デザイン

本研究は，多施設，並行群間，オープンラベル，ランダム化比較試験である

## 6.2. 目標症例数

研究全体の目標症例数：200症例（試験治療群100症例，標準治療群100症例）

症例数の設定根拠は「9.2.目標症例数の設定根拠」参照

## 6.3. 研究期間

研究期間：jRCT初回公表日（厚生労働大臣届日）から2028年3月31日まで

登録期間：jRCT初回公表日（厚生労働大臣届日）から2025年3月31日

参加期間は,同意取得日から最長5週間（スクリーニング期間最長1日間，投与開始日～最終観察日まで最長35日間，うち投与期間最大7日間）

## 6.4. 施設登録及び症例登録・割付方法

施設登録及び症例登録は，登録センター（担当部署：横浜市立大学附属病院 集中治療部）の中央登録制とする．施設登録が完了した研究機関から症例登録が可能となる．施設登録・症例登録については，14項を参照．

6.4.1. 登録センター

担当者： 中村謙介

施設名：横浜市立大学附属病院　集中治療部

TEL：045-787-2800

Mail：nakamura.ken.kl@yokohama-cu.ac.jp

受付時間：　平日9時から17時（土日，祝祭日，年末年始は受付けない）

6.4.2. 施設登録

各実施医療機関の研究責任医師は，認定臨床研究審査委員会での承認後に実施医療機関の管理者の実施に関する承認を得た上で，実施医療機関の管理者の実施承認書（写し）を登録センターに送付する．登録センターはTXPにアカウント発行依頼書を送り，TXPが施設登録および担当医師のユーザー登録を行う．

6.4.3. 症例登録（及び割付）

（1）研究責任医師又は研究分担医師は，候補となる研究対象者から文書による同意を取得後，適格性を判断するために必要な検査等を実施する．その際，同意取得前の検査データ等を使用することも許容する．研究対象者が選択基準を満たし，かつ除外基準のいずれにも該当していないことを確認する．

（2）研究責任者又は研究分担者は,EDC上で症例登録を行う.この際,各研究機関において研究対象者識別コードを付与するが,この研究対象者識別コードは単体で特定の個人を識別することができる情報を含まない任意のコードを用いる．

（3）EDC上で適格性を判定し,症例登録と割付を実施する．

（4）研究責任者又は研究分担者は登録後速やかに,EDC上で割付結果を確認する．

（5）研究責任者又は研究分担者はEDC上でいつでも登録割付結果を閲覧できる．

6.4.4. 割付方法と割付調整因子

登録にあたって対象患者はEDC上でランダムに割り付けられる．ランダム割付に際しては，EDC（TXP Medical株式会社に委託して作成）内でプログラムを使用してブロックランダム化し割付ける．詳細は割付仕様書に記載する．層別化は行わない．

6.4.5. 盲検化の方法

本研究では盲検化しないため，該当しない．

6.4.6. 開鍵（キーオープン）の必要性の判断及び手順

本研究では盲検化しないため，該当しない．

## 6.5. 治療計画

6.5.1. プロトコル治療

6.5.1.1. 試験治療群

ハイネックスリニュートを栄養チューブより経胃持続投与し， 3日間継続する．なお，担当医の判断の下，以降7日目までは継続投与可能とするが，対照製剤は投与しない．8日目以降は，試験製剤・対象製剤のいずれも投与しない．

6.5.1.2. 対照治療群

グルセルナ-REXを栄養チューブより経胃持続投与し， 3日間継続する．なお，担当医の判断の下，以降7日目までは継続投与可能とするが，試験製剤は投与しない．8日目以降は，試験製剤・対照製剤のいずれも投与しない．

なお，登録から試験品投与開始までの期間に制限はなく，医学的に妥当と判断されたタイミングでENを開始する．両群ともに栄養プロトコルとして，投与速度は10ml/h から開始とし，有害事象がなければ，8時間おきに10ml/h 増量する．ただし，投与カロリーが20kcal/kg/日を超えない範囲とし，目標エネルギー投与量は規定せず主治医が決定できる．

[設定根拠] 重症病態における経腸栄養のプロトコルに一般的に導入されている投与速度を参考とした^E5)^

6.5.2. 投与基準

6.5.2.1. 投与開始基準

担当医の判断によるものとし，特に規定を設けない．

6.5.2.2 減量・休薬基準

下記の場合は，投与速度の増量の見合わせ，減量または中止を考慮する（担当医により適宜判断）．

(1)胃内残液200ml/8h以上の場合
（通常診療としての，担当看護師による経鼻胃管の吸引・内容量の記録を参考とする）

(2)下痢が発生した場合

(3)その他，ENによる有害事象を認め，担当医が増量を不適切と判断した場合

6.5.2.3再開基準

休薬基準を設定しないため再開基準は設定しない..

6.5.3. 併用薬・併用療法

- - 項目名の“○○投与群”は実際の薬剤名等に変更する等適切に修正する.
  - 登録後何日以内に試験治療薬の投与を開始する必要があるのかを規定する.
  - 試験治療薬の用法・用量，投与期間を明記する.
  - 遵守すべき事項を記載する（入院が必要な場合は入院期間など）.

6.5.3.1. 併用禁止治療

Primary outcome評価までプロトコル治療以外の経腸栄養製剤は両群ともに使用しない．ただし栄養剤でない薬剤投与は可能とし，静脈栄養は適宜可能とする．またそれ以外の治療に制限は設けない．

6.5.3.2. 併用注意治療

該当なし

6.5.3.3. 併用可能治療

(1) 発熱性好中球減少やその他の感染症に対するG-CSFや抗生剤

(2) 悪心・嘔吐の軽減を目的とした予防的な 5HT3拮抗剤

(3) 止痢剤

(4) 皮膚症状に対するステロイド外用薬

(5) 鎮痛目的のオピオイド製剤

(6) その他，合併症および有害事象に対する対症療法

6.5.4. 個々の研究対象者における研究の中止

6.5.4.1. 中止基準

以下の基準のいずれかに該当する場合は，研究対象者の研究を中止する．

（1）患者または代諾者から同意撤回の申し出があった場合

（2）登録後に選択基準に合致しない又は除外基準に抵触し対象として不適切であることが判明した場合

（3）疾患の症状，所見の悪化により研究の継続が困難な場合

（4）有害事象の発現により研究の継続が困難な場合

（5）研究計画書からの重大な逸脱が発生した場合

（6）その他，研究の継続が好ましくないと研究責任者又は研究分担者が判断した場合

6.5.4.2. 中止手順

（1）研究責任者又は研究分担者は，研究期間中に研究対象者への試験品の投与を中止する場合，当該研究対象者に速やかにその旨を説明し，必要な観察，検査及び評価を行う．

（2）研究責任者又は研究分担者は，症状の悪化及び有害事象の発現等，研究対象者の安全性の問題により当該研究対象者への試験品の投与を中止した場合には研究対象者に対し適切な処置を施す．また，症状（臨床検査値も含む）が試験品投与前もしくはベースラインの状態に回復するまで，又は症状が安定するまでの期間，経過観察を継続し，その転帰を記録する．

（3）投与を中止した日付と理由を症例報告書に記載する．

6.5.4.3. プロトコル治療終了（中止）後の後治療

本研究では該当しない．

## 6.6. 研究全体の中止

下記に該当した場合は研究全体を中止する．研究責任医師は，研究を中止した場合には，研究対象者に中止したことを速やかに通知し，適切な医療の提供やその他の必要な措置を講ずる．研究代表医師は各実施医療機関の研究責任医師及び認定臨床研究審査委員会，実施医療機関の管理者に報告するとともに，中止日より10日以内に，統一書式11中止通知書を作成し，認定臨床研究審査委員会に通知する．また，研究代表医師は，様式第四 特定臨床研究中止届書を作成し，厚生労働大臣に届け出る．なお，研究を中止した場合であっても，主要評価項目報告書，総括報告書及びその概要については適切に作成し，それらの作成と研究が終了するまでの間，定期報告及び疾病等報告を実施する．

(1) 予測できない重篤な疾病等が発生し，研究対象者全体への不利益が懸念される場合

(2) 中間解析の結果等で有効性や優位性などが無いと判断された場合

(3) 法及び関連法令又は研究計画書に対する重大な違反／不適合が判明した場合

(4) 倫理的妥当性もしくは科学的合理性を損なう，又は損なう恐れのある事実を得た場合

(5) 研究対象者に対する重大なリスクが特定された場合

(6) 症例登録の著しい遅れ，研究計画書からの逸脱の頻発などの理由により，研究の完遂が困難と判断した場合

(7) 実施医療機関の管理者や認定臨床研究審査委員会に中止要請や勧告を受けた場合

(8) 厚生労働大臣に中止要請や勧告を受けた場合

# **7. 観察・検査・調査・評価項目**

## 7.1. スケジュール表

| 実施 | | スクリーニング | 投与 | 投与期間 | | | 後観察 | | 終了時 | 中止時 |
| --- | --- | --- | --- | --- | --- | --- | --- | --- | --- | --- |
|  | | 期間 | 開始日  Day0 | 0週  Day1 | 0週  Day2 | 0週  Day3 | 1週  Day7 | 2週  Day14 | 4週  D28 |  |
| 許容範囲（日） | | ICU入室～0 | 0 | 0 | 0 | 0 | ±2 | ±2 | ＋7 | ±3 |
| 同意取得・登録 | | ○ |  |  |  |  |  |  |  |  |
| 患者背景の確認 | | ○ |  |  |  |  |  |  |  |  |
| 試験品投与 | |  |  | 🡨--------------------🡪 | | | 注1 |  |  |  |
| SOFAスコア | | ○ |  |  |  |  |  |  |  |  |
| 栄養アセスメント情報 | | ○ |  |  |  |  |  |  |  |  |
| 消化器症状 | |  | ○ | ○ | ○ | ○ |  |  |  |  |
| 排便所見 | |  | ○ | ○ | ○ | ○ |  |  |  |  |
| 下剤投与の確認 | |  | ○ | ○ | ○ | ○ |  |  |  |  |
| 血液保存　注2 | |  | ○ |  |  | ○ | ○ |  |  |  |
| 糞便保存　注2 | |  | ○ |  |  | ○ | ○ |  |  |  |
| 臨  床  検  査 | 血液学的  検査 | ○ | ○ | ○ |  | ○ | ○ | ○ |  | ○ |
|  | 血液生化学  検査 | ○ |  |  |  |  | ○ | ○ |  | ○ |
| 死亡確認 | |  |  |  |  |  |  |  | ○ |  |
| ICU入室期間情報 | |  |  |  |  |  |  |  | ○ |  |
| 有害事象の観察 | |  | 🡨------------------------------------------------------------🡪 | | | | | | | ○ |

1. 投与期間のDay 3以降も臨床的判断の下,投与可能
2. オプションとして同意取得された被験者のみ

## 7.2. 実施スケジュール時期と評価項目

7.2.1. スクリーニング期間

1. 研究対象者背景

- 性別，年齢，身長，体重，既往歴，合併症
- 同意取得日
- 原疾患の診断名，疾患カテゴリー（感染症，心不全，呼吸不全，脳卒中，腎・代謝疾患，術後，CPA蘇生後，外傷，その他）

1. SOFAスコア

- 呼吸器ポイント
- 新血管系ポイント
- 腎臓系ポイント
- 肝臓系ポイント
- 凝固系ポイント
- 中枢神経系ポイント

1. 栄養アセスメント情報（MUST）
2. 血液学的検査（WBC, リンパ球数）
3. 血液生化学検査（CRP，アルブミン，プレアルブミン，総/HDL/LDLコレステロール値，中性脂肪値）

7.2.2. 0日（投与開始前）

1. 消化器症状（嘔気・嘔吐，腹痛，3日間のGRV/8hの平均，腸管虚血イベント，イレウス所見，下血の有無）
2. 排便所見（Bristol Scale），(200 g/day, or 300ml/day)の下痢の有無， (1日3回以上の水溶性下痢)の有無
   排便所見は，担当看護師が排泄処理時に随時評価し，診療録へ記載する（1～3日目も同様とする）
3. 下剤投与の有無の確認
4. 血液学的検査（WBC, リンパ球数）
5. 有害事象
6. Day0（介入製剤投与前）の血液検体で血漿と全血を保存（オプションとして同意取得された被験者のみ）
7. Day0（介入製剤投与前）の糞便を保存（オプションとして同意取得された被験者のみ）

7.2.3. 1日（投与開始後24時間 時点）

1. 消化器症状（嘔気・嘔吐，腹痛，3日間のGRV/8hの平均，腸管虚血イベント，イレウス所見，下血の有無）
2. 排便所見（Bristol Scale），(200 g/day, or 300ml/day)の下痢の有無， (1日3回以上の水溶性下痢)の有無
3. 下剤投与の有無の確認
4. 血液学的検査（WBC, リンパ球数）
5. 有害事象

7.2.4. 2日（投与開始後48時間 時点）

1. 消化器症状（嘔気・嘔吐，腹痛，3日間のGRV/8hの平均，腸管虚血イベント，イレウス所見，下血の有無）
2. 排便所見（Bristol Scale），(200 g/day, or 300ml/day)の下痢の有無， (1日3回以上の水溶性下痢)の有無
3. 下剤投与の有無の確認
4. 有害事象

7.2.5. 3日（投与開始後72時間 時点）

1. 消化器症状（嘔気・嘔吐，腹痛，3日間のGRV/8hの平均，腸管虚血イベント，イレウス所見，下血の有無）
2. 排便所見（Bristol Scale），(200 g/day, or 300ml/day)の下痢の有無， (1日3回以上の水溶性下痢)の有無
3. 下剤投与の有無の確認
4. 血液学的検査（WBC, リンパ球数）
5. 有害事象
6. day3の血液検体で血漿と全血を保存（オプションとして同意取得された被験者のみ）
7. day3の糞便を保存（オプションとして同意取得された被験者のみ）

7.2.6 後観察（1週，2週）

1. 血液学的検査（WBC, リンパ球数）
2. 血液生化学検査（CRP，アルブミン，プレアルブミン，総/HDL/LDLコレステロール値，中性脂肪値）
3. day7（±1）の血液検体で血漿と全血を保存
4. day7（±1）の糞便を保存
5. 有害事象

7.2.7.終了時（4週）

1. 試験品投与開始後28日死亡の有無
2. ICU入室期間情報

- ICU入室日数
- 入院日数
- 人工呼吸器の有無と日数
- 血液浄化の有無と日数
- 体外式膜型人工肺の有無と日数
- 持続昇圧薬投与有無（昇圧薬はドパミン，ドブタミン，ノルエピネフリン，エピネフリン，バソプレシンのいずれか1つでも使用している場合とする）
  試験品投与後3日間での投与の有無を収集（具体的薬品名の収集は行わない）
- 持続鎮静薬投与の有無（鎮静薬はプロポフォール，ミダゾラム，デクスメデトミジン，チオペンタール，ケタミンのいずれか1つでも使用している場合とする）
  試験品投与後3日間での投与の有無を収集（具体的薬品名の収集は行わない）
- 持続オピオイド投与の有無（オピオイドはフェンタニル，レミフェンタニル，モルヒネのうちいずれか1つでも使用している場合とする）
  試験品投与後3日間での投与の有無を収集（具体的薬品名の収集は行わない）
- 消化管機能改善薬の投与の有無
  試験品投与後3日間での投与の有無を収集（具体的薬品名の収集は行わない）
- 試験品投与開始後28日目時点でのBarthel Index (それまでに退院した場合は退院時のBarthel Indexとする)
- EN投与開始後３日目と７日目の時点でのEN失敗の有無（有りの場合，その理由の選択：下痢，嘔吐，腸管虚血，その他のEN有害事象，抜管，食事開始，検査・部屋移動，その他のEN有害事象でない理由）
- EN投与期間
- EN開始後はじめの7日間の，ENと静脈栄養にわけた毎日のエネルギー投与量kcal/day，タンパク質投与量g/day
- 併用禁止薬の使用の有無

1. 有害事象

7.2.8.中止時

1. 血液学的検査（WBC, リンパ球数）
2. 血液生化学検査（CRP，アルブミン，プレアルブミン，総/HDL/LDLコレステロール値，中性脂肪値）
3. 有害事象

# **8. 評価項目**

## 8.1. 主要評価項目

試験品投与開始3日以内の下痢（Bristol Scale 5，6，7で定義）の発生率

（上記期間で，一度でも下痢が確認されればイベント発生とする）

（6.5.2.2 減量・休薬基準に従って減量・休薬した場合も，定義を満たせばイベント発生とする）

【設定根拠】EN時の下痢はコントロールに難渋することも多く，下痢の有無は関心の高い事項である．

通常のEN製剤の腸管滞留時間が最も吸収に時間のかかる脂質で24時間以上かかるため，EN開始して腸管全体にEN製剤が浸透するのに24－48時間，EN製剤に伴う下痢であればその後24時間以内に発生しうると考えて，EN製剤開始3日以内の下痢の発生を見極めれば十分と考えられる．

下痢の指標は過去研究での引用および近年のレビューによる推奨を根拠にBristol Scaleを用い^11)^，臨床上で有意な影響があるScale 5以上を下痢と定義する．

## 8.2. 副次評価項目

・試験品投与開始1，2，3日目のBristol Scale，(200 g/day, or 300ml/day)の下痢の有無， 1日3回以上の水溶性下痢（Bristol Scale 7）の有無

・試験品投与開始後はじめの1週間の下痢（Bristol Scale 5，6，7で定義）発生の有無，（カルテレビューにより補完）

・試験品投与開始3，7日目のEN失敗率： 各群に割付られた被験者に対して，下痢，嘔吐，腸管虚血，その他のEN有害事象の理由によってEN失敗となった被験者の割合（抜管，食事開始，検査・部屋移動，その他のEN有害事象でない理由はEN失敗であっても含めない）

・ENの投与期間

・試験品投与開始後はじめの7日間の，ENと静脈栄養にわけた毎日のエネルギー投与量kcal/day，タンパク質投与量g/day，なおエネルギー量には5％ブドウ糖液より薄いエネルギー密度の静脈栄養およびプロポフォールの栄養を含まない計算とする

・試験品投与開始後28日生存率

・ICU入室日数

・入院日数

・人工呼吸日数

・栄養評価項目：WBC, リンパ球数，CRP，アルブミン，プレアルブミン，総/HDL/LDLコレステロール値，中性脂肪値（7日目，14日目）

・試験品投与開始後28日目時点でのBarthel Index (それまでに退院した場合は退院時のBarthel Indexとする)

## 8.3. 安全性評価項目

安全性評価項目として，有害事象および副作用を収集する．

・腸管不耐イベントの発生率

　：消化器症状（嘔気・嘔吐，腹痛，3日間のGRV/8hの平均，腸管虚血イベント，イレウス所見，下血の有無）

腸管虚血の定義：以下のいずれか（CT等の画像検査により腹腔動脈または上腸間膜動脈または下腸間膜動脈の阻血が示唆された場合，内視鏡で大腸虚血を認めた場合，手術所見で腸管虚血を認めた場合）^15)^

・試験品投与開始後28日以内の感染イベントの発生率：院内肺炎，菌血症，カテーテル関連感染，尿路感染，軟部組織感染，その他の感染

院内肺炎の定義：胸部レントゲン写真での浸潤影および以下2つ（>38℃の発熱，白血球数<4000または>11000/μl，膿性喀痰）^16)^

# 9. 統計解析

## 9.1. 解析対象集団

解析対象集団は，以下のように定義する．主たる解析対象集団はFASとし，有害事象の解析はSASとする．

9.1.1. 最大解析対象集団（full analysis set：FAS）

試験治療が少なくとも1回以上行われ，primary outcomeの観察が行われた集団をFASとする．

9.1.2. 安全性解析対象集団（safety analysis set：SAS）

本研究に登録され，少なくとも１度は試験品を投与された集団をSASとする．

## 9.2. 目標症例数の設定根拠

研究全体の目標症例数：200例 （試験品群100例，対照群100例）

【設定根拠】

過去の後ろ向き研究のデータを参考^10)^に，検証試験としての本研究の各群の下痢の発生割合を見積もった．対照群（グルセルナ）の下痢を先行研究のtraditional liquid EN（メイバランス）よりも脂質含有が多いため10％程度多く見積もって50％とし，介入群（ハイネックスリニュート）の下痢を先行研究のPectin-containing liquid EN（ハイネックスイーゲル，ハイネックスリニュートと同じくペクチンが含まれるが脂肪含有が少なく濃度も薄いもの）よりわずかに多く30％と見積もった．

以上より，介入群と対照群の下痢発生割合を30％と50％と仮定し，両側有意水準5％の下でPearson’s chi-square testを適用すると，検出力を80％保持するために必要な症例数は各群と93例と算出された．若干の脱落例等を考慮して，目標症例数は各群100例，計200例とした．

## **9.3. 統計解析方法**

**9.3.1. 主要評価項目の解析**

**9.3.1.1 主要な解析**

FASを対象に,以下の集計解析を行う.

度数集計および信頼区間（Wilson score interval）を算出する.また,Pearson’s chi-square testを用いて群間比較を行う.

**9.3.1.2. 副次的な解析**

　FASを対象に，以下のサブグループ解析を行う.

・性別（男性／女性）

・年齢（70歳未満／70歳以上）

・BMI（20未満／20以上30未満／30以上）

・SOFAスコア（各項目で1つでも2点未満がある／それ以外）

・疾患カテゴリー（感染症／心不全／呼吸不全／脳卒中／腎・代謝疾患／術後／CPA蘇生後／外傷／その他）

[設定根拠] 重症患者では下痢は多くの要因を受けておこる^E2)^ため，特に試験薬が有効なサブグループを特定することは有用であり今後のプラクティスに活用できる

**9.3.2. 副次評価項目の解析**

FASを対象に,以下の集計解析を行う. また, 群間差の信頼区間（Pearson’s Chi-Square）も算出する.

**9.3.2.1. EN失敗率,投与開始後28日生存率**

度数集計および信頼区間（Wilson score interval）を算出する.

**9.3.2.2. ICU入室日数,入院日数,人工呼吸日数**

要約統計量を算出する．

**9.3.2.3. その他の副次評価項目**

2値データに対しては度数を集計し,連続値に対しては要約統計量を算出する.

なお,以上の集計結果を参考にして,事後解析としてサブグループ解析などを検討する.

**9.3.3. 安全性解析**

SASを対象に,有害事象および副作用に対して,以下の集計を行う.

発現例数の度数を集計する.また,事象別,重症度別に発現例数の度数を集計する. 加えて，腸管不耐イベントおよび試験品投与開始後28日以内の感染イベントに対して，それぞれ度数を集計する．

## **9.4. 中間解析**

本研究では中間解析は実施しない.

## **9.5. 一般的事項**

- 検定における有意水準は原則両側5％とし,信頼区間は両側95％信頼区間を算出する
- 連続変数に対する要約統計量は,例数,平均値,標準偏差,最小値,最大値,中央値,第一四分位点,第三四分位点を算出する.症例報告書に入力された小数点以下の桁数+2桁を四捨五入して+1桁で表示する.最大値,最小値について,症例報告書に入力された小数点以下の桁数と同桁で表示する
- カテゴリー変数に対する度数集計は,頻度と割合を算出する.割合の桁数は,小数第1位未満を四捨五入して小数第1位までの％表示とする

# **10. 疾病等の取り扱いについて**

## 10.1. 疾病等の定義

疾病等とは，特定臨床研究の実施に起因するものと疑われる疾病，障害若しくは死亡又は感染症であり，意図しない徴候，臨床検査値の臨床的に有意な変動，症状，合併症の悪化を含む．

本研究では治療開始日から後観察期間終了までの間に特定臨床研究の実施に起因するものと疑われる事象を疾病等として取り扱い，重篤な疾病等及び全gradeの疾病等についてのデータを収集する．

## 10.2. 疾病等の評価

疾病等の程度はCommon Terminology Criteria for Adverse Event v5.0に準じ，以下のように評価する．なお，実際治療したかどうかではなく必要性で判定する．

Grade1：軽度（疾病等に対して，治療介入の必要がない）

Grade2：中等度（疾病等に対して，外来薬物治療などの治療介入が必要）

Grade3：重度（疾病等に対して，入院での治療が必要）

Grade4：生命を脅かす，又は活動不能となる

Grade5：死亡

プロトコル治療との因果関係は以下の5つに分類する

1. あり：明確に疾病等が，プロトコル治療により生じた/重症化したことが明らかで，原病の増悪や他 　　　　の要因（併存症，他の薬剤・治療，偶発症）による可能性がほとんどないと判断される．
2. おそらくある：疾病等が，原病の増悪や他の要因（併存症，他の薬剤・治療，偶発症）により生じた/重症化した可能性はありそうになく，プロトコル治療によると考える方が合理的（plausible） と判断される．
3. ありうる： 疾病等が，プロトコル治療により生じた/重症化したのか，原病の増悪や他の要因（併存症，他の薬剤・治療，偶発症）によるのかいずれとも決めがたい．
4. ありそうにない：疾病等が，プロトコル治療により生じた/重症化した可能性はありそうになく，原病の増 悪や他の要因（併存症，他の薬剤・治療，偶発症）によると考える方が合理的（plausible）と 判断される．
5. なし：疾病等が，原病の増悪や他の要因（併存症，他の薬剤・治療，偶発症）により生じた/重症化したことが明らかで，プロトコル治療による可能性がほとんどないと判断される．

上記分類のうち，①～③のいずれかと判断された場合は「因果関係あり」とし，④及び⑤のいずれかと判断された場合は 「因果関係なし」とする．

以下のいずれかに該当するものを「重篤な疾病等」とする

(1) 死亡

(2) 死亡につながるおそれのある疾病等

(3) 治療のために医療機関への入院又は入院期間の延長が必要とされる疾病等

(4) 障害

(5) 障害につながるおそれのある疾病等

(6) (3)から(5)まで並びに死亡及び死亡につながるおそれのある疾病等に準じて重篤である疾病等

(7) 後世代における先天性の疾病又は異常

## 10.3. 予測できる疾病等

経腸栄養剤は医薬品ではないので有害事象のデータは存在しないが，一般的なEN同様に，消化器症状（下痢，嘔気・嘔吐，腹痛，腸管虚血，イレウス，下血）が予想される．海外における早期ENを検討したNUTRIREA-2試験では，EN群において下痢（36%），嘔吐(34%)，腸管虚血（2%），イレウス（1%）で認めた．^17)^

よってこれらの消化管症状のうち，Grade2以上に相当するものとして腸管虚血は必ず有害事象と扱い報告する．腸管虚血は以下のいずれかと定義する：腸管に血液を供給する主要動脈（上腸間膜動脈，下腸間膜動脈，または腸骨動脈）のいずれかに血流がなく，画像検査（CT血管造影，血管造影，または磁気共鳴血管造影）で腸管壁の障害を示す証拠があること，Favier分類システムによる大腸虚血の内視鏡検査基準が存在すること（I期， 点状出血，II期，点状出血および表在性潰瘍，III期，壊死性潰瘍およびポリープ状病変），および手術中の腸管虚血の所見を認めること．

## 10.4. 疾病等が発現した場合の措置

10.4.1. 研究対象者への措置

研究責任医師又は研究分担医師は，疾病等が発生した場合，研究対象者の安全確保のため必要に応じ研究対象者に対し，治療及び試験品投与の中止等，適切な措置を講じる．治療等が必要となった場合は，その旨を研究対象者に伝える．

研究責任医師又研究分担医師は，試験品終了時の最終観察時点で疾病等が継続している場合は，それ以降もベースライン値の状態（Grade）に回復するまで，又は臨床的に安定するまで追跡調査を実施する．

10.4.2. 評価及び記録

研究責任医師又研究分担医師は，発現した疾病等について，原資料（診療録等）に疾病等名，発現日，重症度，重篤・非重篤の別，処置・治療の内容，転帰（回復した場合は回復時期，症状が固定した場合はその時期）を記載する．

10.4.3. 重篤な疾病等の報告

重篤な疾病等の発生を知った場合，実施医療機関の研究責任医師は，速やかに当該実施医療機関の管理者に報告した上で，研究代表医師に通知する．研究代表医師は，当該疾病等について実施計画に記載された認定臨床研究審査委員会に報告すると同時に，厚生労働大臣（医薬品医療機器総合機構：PMDA）へ報告し，試験品の製造販売をし，又はしようとする医薬品等製造販売業者に情報提供を行う．研究代表医師は，「表10.4.3 　重篤な疾病等が発生した場合の報告期限」に従い，疾病等の報告を行い，報告期限を遵守する．

表10.4.3　重篤な疾病等が発生した場合の報告期限

| 疾病等 | 予測可能性 | 管理者及び委員会への報告期限 | PMDA  への報告期限 |
| --- | --- | --- | --- |
| - 1. 死亡 | 予測できない^注１）^ | 7日以内 | 7日以内 |
|  | 予測できる | 15日以内 | 定期報告^注２）^ |
| - 1. 死亡につながるおそれのある疾病等 | 予測できない | 7日以内 | 7日以内 |
|  | 予測できる | 15日以内 | 定期報告 |
| - 1. 治療のために医療機関への入院又は入院期間の延長が必要とされる疾病等 | 予測できない | 15日以内 | 15日以内 |
|  | 予測できる | 定期報告 | 定期報告 |
| - 1. 障害 | 予測できない | 15日以内 | 15日以内 |
|  | 予測できる | 定期報告 | 定期報告 |
| - 1. 障害につながるおそれのある疾病等 | 予測できない | 15日以内 | 15日以内 |
|  | 予測できる | 定期報告 | 定期報告 |
| - 1. 上記の①から⑤までに準じて重篤である疾病等 | 予測できない | 15日以内 | 15日以内 |
|  | 予測できる | 定期報告 | 定期報告 |
| - 1. 後世代における先天性の疾病又は障害 | 予測できない | 15日以内 | 15日以内 |
|  | 予測できる | 定期報告 | 定期報告 |

注1)： 「予測できない」疾病等の定義：疾病等の内、医薬品等の概要を記載した文書に記載されていないもの、あるいは記載されていてもその性質や重症度が記載内容と一致しないものをいう。＜研究計画書に定義が記載されている場合はここでの定義は不要となる。＞

注2)： 別紙様式３「定期報告書」により、特定臨床研究の実施状況の報告に合わせて法第１３条に基づく疾病等の報告　件数を報告する。

10.4.4. 疾病等の報告

疾病等の発生を知った場合，実施医療機関の研究責任医師は，１年ごとの定期報告を行うときに当該実施医療機関の管理者に報告した上で，研究代表医師に通知する．研究代表医師は，当該疾病等について実施計画に記載された認定臨床研究審査委員会に報告する．また，研究代表医師は，速やかに他の研究責任医師に情報提供し，当該他の研究責任医師は，速やかに当該情報提供の内容を実施医療機関の管理者に報告する．研究代表医師は，実施計画を厚生労働大臣に提出した日から起算して，1年ごとに，当該期間満了後2月以内に疾病等の報告を行い，報告期限を遵守する．

10.4.5. 不具合報告

該当なし

## 10.5. 疾病等を除く有害事象について

10.5.1.　有害事象の定義

有害事象とは，因果関係の有無を問わず，研究対象者に生じたすべての好ましくない又は意図しない傷病若しくはその徴候（臨床検査値の異常を含む．）をいう．研究期間中に既存の疾患（原病は含まない）が増悪した場合も含む．ただし，疾病等に該当する場合は，10.1.から10.3.に従うものとする．

10.5.2.　 有害事象の評価

有害事象の評価については，10.2.を準用する．

10.5.3.　研究対象者への措置

研究対象者への措置については，10.4.1を準用する．

10.5.4.　評価及び記録

評価及び記録については，10.4.2を準用する．

10.5.5.　重篤な有害事象の報告

多施設共同臨床研究の各実施医療機関において疾病等を除く重篤な有害事象が発生した場合には，当該実施医療機関の研究責任医師は速やかに研究代表医師に報告する．研究代表医師は，必要に応じ共同研究を実施している各実施医療機関の研究責任医師に通知する．なお，疾病等を除く重篤な有害事象について，研究責任医師は実施医療機関の管理者に報告する必要はないものとする．

# **11. データマネージメント**

本研究では，データセンター担当者がデータマネジメントを実施する..電子症例報告書に入力されたデータで，データモニタリングによって確認された外れ値や異常値等は，クエリとして電子症例報告書上で確認及び修正の依頼を行う..データセンターのデータ固定後に，統計解析責任者に対して固定データが提供される..詳細に関しては，データマネジメント計画書に規定する..

# **12. 効果安全性評価委員会**

本研究では効果安全評価委員会は設置しない．

# **13. 研究計画書の遵守，変更及び不適合の管理（研究計画書からの逸脱等）について**

## 13.1. 研究計画書の遵守

研究責任医師又は研究分担医師は，研究代表医師の事前の合意及び実施計画に記載のある認定臨床研究審査委員会の審査に基づく文書による事前の承認を得ることなく，研究計画書に適合しないこと（すなわち研究計画書からの逸脱又は変更）を行ってはならない．

## 13.2. 研究計画書の変更

13.2.1. 研究計画書の変更手順

研究計画書の内容を変更する場合は，以下の手順により行う．

1）研究代表医師は，研究計画書の変更が必要と判断した場合は，研究責任医師に研究計画書変更案及びその他必要な資料・情報を提供する．

2）研究代表医師は，研究責任医師が前項により提供された研究計画書変更案及びその他の資料・情報を十分検討し，研究代表医師と協議するのに必要な時間を研究責任医師に与える．

3）研究代表医師は，研究責任医師の合意が得られた後に，変更内容を記載した文書及び変更した研究計画書から必要に応じ実施計画を作成する．

4）研究代表医師は，変更した研究計画書等について実施計画に記載のある認定臨床研究審査委員会の意見を聴き，承認を得る．

5）研究代表医師は，審査結果を踏まえ研究責任医師に報告を行い，実施医療機関の管理者に実施の承認を得る．

## 13.3. 不適合の管理（研究計画書不遵守等）

1）研究責任医師又は研究分担医師は，不適合であることを知った時は，速やかに実施医療機関の管理者に報告するとともに，これを研究代表医師に通知する．

2）研究代表医師は，研究対象者の人権，安全性及び研究の進捗並び結果の信頼性に影響を及ぼす重大なもの（例えば，選択・除外基準や中止基準，併用禁止療法等の不遵守をいう．）が判明した場合においては，重大な不適合として，速やかに実施計画に記載のある認定臨床研究審査委員会の意見を聴く．

3）重大な不適合には，臨床研究の対象者の緊急の危険を回避するためその他医療上やむを得ない理由により研究計画書に従わなかったものについては含まない．

# **14. 倫理的事項**

## 14.1 遵守すべき諸規則

本研究は，ヘルシンキ宣言に基づいた倫理原則に則り，臨床研究法，同法施行規則，個人情報保護法及びその他関連通知に従って実施する．研究責任医師及び研究分担医師は，本研究計画書を遵守してプロトコル治療を実施する．

## 14.2. 個人情報等の取扱い

本研究では患者を登録する際に，研究対象者識別コードを付与する．研究対象者識別コードは，イニシャルやカルテID等のような特定の個人を識別できる情報とは無関係の数字記号等で構成され，症例登録票や症例報告書等の本研究に関する書類を作成する際には研究対象者識別コードを使用することで個人を識別できない状態にして取り扱う．研究責任医師は，個人を識別できない状態にした試料・情報と，研究対象者を識別することができるように研究対象者の氏名やカルテID等の情報が記載された情報を紐づける，個人情報を管理する表（以下，対応表）を作成し，外部に漏洩することがないよう厳重に保管管理を行う．（管理責任者：各施設の研究責任医師）

本研究は横浜市立大学附属病院を含む複数施設で実施する多施設共同研究であり，各医療機関で個人を識別できない状態にした試料・情報と，研究対象者を識別することができるように研究対象者の氏名やカルテID等の情報が記載された情報を紐づける，個人情報を管理する表（以下，対応表）を適切に管理することとし，実施医療機関外への提供は行わない．研究対象者の症例登録票，症例報告書，検査データ等を当該研究機関外に提供する際には，研究対象者識別コード又は登録番号を用いる（どの研究対象者の試料・情報であるかが直ちに判別できないよう，加工又は管理されたものに限る）．本研究における個人情報等は，本研究計画書のほか，各実施医療機関における臨床研究に関する個人情報等の取扱いに関する手順書を遵守して取り扱う．

## 14.3. 研究参加に伴い研究対象者に予期される利益及び不利益等

14.3.1. 予期される利益

本研究参加により研究対象者に直接の利益は生じない．研究参加により将来の医療の進歩に貢献できる可能性がある．

14.3.2. 予期される不利益

本研究で用いる試験品は試験対象とする病態への効能・効果に関する薬事承認はなく、保険適用されていないが、濃厚流動食品として一般的に保険適用されている。日常診療に比して，研究対象者が本研究に参加することで経済上の負担はない．本研究に参加する事で研究対象者に起こり得る疾病等は，「10.3. 予測できる疾病等」に記載した．研究責任医師又は研究分担医師は，疾病等が発生した場合，「10.4. 疾病等が発現した場合の措置」に従い適切に対処する．

## 14.4. 研究対象者に係る研究結果（偶発的所見を含む．）の取扱い

本研究で研究対象者の健康，子孫に受け継がれ得る遺伝的特徴等に関する重要な知見が得られる可能性はないが，実施する検査等により，研究対象者の健康に重大な影響を与える情報（偶発的所見を含む）を入手した場合は，研究責任医師又は分担医師が研究対象者に説明し，治療や処置を行う等の適切な措置を講じる．また，研究対象者個別における研究参加に伴う結果は診療の中で研究対象者本人に説明する．

## 14.5. 原資料等の閲覧

本研究では，研究責任医師及び実施医療機関が，当該臨床研究に関連するモニタリング，監査並びに認定臨床研究審査委員会及び規制当局の調査の際に，原資料等の全ての臨床研究関連記録を直接閲覧に供する．

# **15. インフォームド・コンセントを受ける手続**

研究責任医師及び分担医師は，患者を登録する前に実施計画に記載のある認定臨床研究審査委員会で承認され実施医療機関の管理者の許可を得た最新の同意・説明文書を用いて以下の事項を十分に説明し患者が内容を十分理解したことを確認した後，参加の同意を文書により取得する．各施設の研究責任医師が必要と判断した場合，施設ごとに説明文書・同意書を変更することができるが，研究代表医師に提出を行い，実施計画に記載のある認定臨床研究審査委員会の承認を得る．研究への参加の継続について臨床研究の対象者又は代諾者の意思に影響を与える可能性のある情報が得られたときは，速やかに説明文書を改訂する．

インフォームド・コンセントを受ける際に研究対象者等に対し説明すべき事項は，原則として以下のとおりとする．ただし，認定臨床研究審査委員会の意見を受けて実施医療機関の管理者が許可した事項については，この限りでない．

１）　実施する特定臨床研究の名称，当該特定臨床研究の実施について実施医療機関の管理者の承認を受けている旨及び厚生労働大臣に実施計画を提出している旨

２）　実施医療機関の名称並びに研究責任医師の氏名及び職名（特定臨床研究を多施設共同研究として実施する場合にあっては，研究代表医師の氏名及び職名並びに他の実施医療機関の名称並びに当該実施医療機関の研究責任医師の氏名及び職名を含む．）

３）　特定臨床研究の対象者として選定された理由

４）　特定臨床研究の実施により予期される利益及び不利益

５）　特定臨床研究への参加を拒否することは任意である旨

６）　同意の撤回に関する事項

７）　特定臨床研究への参加を拒否すること又は同意を撤回することにより不利益な取扱いを受けない旨

８）　特定臨床研究に関する情報公開の方法

９）　特定臨床研究の対象者又はその代諾者（以下「特定臨床研究の対象者等」という．）の求めに応じて，研究計画書その他の特定臨床研究の実施に関する資料を入手又は閲覧できる旨及びその入手又は閲覧の方法

10） 特定臨床研究の対象者の個人情報の保護に関する事項

11） 試料・情報の二次利用について

12）　試料等の保管及び廃棄の方法

13） 特定臨床研究に対する臨床研究法施行規則第21条第１項各号に規定する関与に関する状況

14） 苦情及び問合せへの対応に関する体制

15） 特定臨床研究の実施に係る費用に関する事項

16） 他の治療法の有無及び内容並びに他の治療法により予期される利益及び不利益との比較

17） 特定臨床研究の実施による健康被害に対する補償及び医療の提供に関する事項

18） 特定臨床研究の審査意見業務を行う認定臨床研究審査委員会における審査事項その他当該特定臨床研究に係る認定臨床研究審査委員会に関する事項

19）その他特定臨床研究の実施に関し必要な事項

## 15.1. 研究対象者等及びその関係者からの相談等への対応

研究対象者等やその関係者からの相談には，研究責任医師又は研究分担医師が対応する．対応の方法が不明の場合には，相談内容に合わせて研究事務局と協議の上，対応する．

## 15.2. 代諾者等からインフォームド・コンセントを受ける場合

本研究はICU入室患者が対象となっているため、直接本人から文章による同意を得られない可能性がある。その場合は、代諾者として臨床研究の対象者の父母、兄弟姉妹、子・孫、祖父母、同居の親族またはそれら近親に準ずると考えられる者から同意を得ることとし、同意に関する記録とともに代諾者と患者との関係を示す記録を残す。

## 15.3. インフォームド・アセントを得る場合

本研究では成人年齢以上の患者を対象としているため該当しない．

## 15.4. 同意後に同意撤回を受ける場合

　　研究参加の同意を得た後，患者本人から研究参加への同意を取り消す申し出があった場合は同意撤回とする．同意撤回においては，文書のみならず口頭等意思が確認できる手段を可能とし，同意撤回により不利益が生じないよう配慮する．

同意撤回書または口頭や手紙などによる同意撤回の意思表示の際は，診療録に記録し研究対象者の安全を最大限に配慮し可能な中止時検査や安全性の調査を行うよう努める．

なお，同意撤回とは，研究参加への同意の撤回を意味し，プロトコル治療継続の拒否(下記①)とは区別する．同意の撤回が表明された場合には，下記②か③のいずれであるかを明確にし，すみやかに研究事務局に連絡する．

②同意撤回の場合は，以降のプロトコルに従ったフォローアップの依頼を中止する．③の場合は，全同意撤回の患者データは，データの取り扱いによって解析対象から除く．

当該患者のフォローアップの依頼の中止および患者データ削除の手順は別途，手順書に定めることとし，それぞれの作業が完了したことを，研究代表医師に報告する．

①患者拒否：以降のプロトコル治療継続の拒否(フォローアップは続ける)．

②同意撤回：研究参加への同意を撤回し，以後のプロトコルに従った治療，フォローアップのすべてを不可とすること．同意撤回以前のデータの研究利用は可．

③全同意撤回：研究参加への同意を撤回し，登録時の情報を含む研究参加時点からのすべてのデータの研究利用を不可とすること．

## 15.5. 特定臨床研究対象者等に対する同意取得が不要な場合

本研究では該当しない．

# **16. 試料・情報の保管及び廃棄の方法**

［試料の保管］

本研究で採取する試料のうち臨床検査のための測定を行った残りの試料・血清は,検査機関・病院・大学の手順に従って速やかに廃棄する.

オプションとして同意取得された被験者のみ，Day0（投与開始前）Day3およびDay7に血漿，全血及び糞便を採取し－80℃で保存する．同検体は採取を行った実施医療機関で匿名化をした後に，別途定める手順に従って横浜市立大学麻酔科学教室に搬送され研究終了後5年間にわたって保存し，二次解析に使用する．保存後には病院の手順に従って適切に廃棄する．

［情報の保管］

研究責任者は，下記に掲げる本研究に関する文書および記録を施錠可能な保管庫で厳重に保管管理する．電子データで保管する場合は，パスワードを設定した上で，院内LANやインターネットから独立したパソコンまたはUSBメモリ等の電磁的記録媒体にて保管し，使用していない時は施錠可能な保管庫で厳重に保管管理する．保管期間は，研究の終了について報告された日から5年間もしくは当該研究の結果の最終の公表について報告された日から3年を経過した日のいずれか遅い日までの期間とする（が，個人が特定できないよう加工した情報については二次利用の可能性があるため保管期間終了後も期間を定めず保管する）．

保管方法は情報の種類に応じて以下のとおり管理する．

保管する情報の種類

・研究対象者の個人が特定できる情報を含む情報（研究対象者の同定及び照会のため，研究対象者識別コードから研究対象者を特定することができる個人情報を管理する表（以下「対応表」という．），同意書，同意撤回書等）

・研究対象者の個人が特定できないよう加工した情報

・研究に関する資料（研究計画書等の審査書類等）

それぞれ以下の通り保管する．

保管期間を経過した文書及び記録は，個人情報や機密情報の漏洩がないように細心の注意を払い廃棄する．紙媒体はシュレッダーにて裁断し廃棄する．その他の媒体に関しては，個人を識別できない状態にした上で，削除等の適切な方法により廃棄する．

1）研究対象者を特定する事項（対応表）

2）研究対象者に対する診療及び検査に関する事項

3）特定臨床研究への参加に関する事項（症例登録票）

4）研究計画書

5）実施計画

6）説明文書・同意書・同意撤回書

7）同意書（署名ありの原本）

8）症例報告書（写）

9）認定臨床研究審査委員会へ提出した書類

10）認定臨床研究審査委員会の結果通知書及び実施医療機関の管理者の実施承認書

11）モニタリング及び監査（監査を実施する場合）に関する文書

12）特定臨床研究の実施に係る契約書

（医薬品等製造販売業者又はその特殊関係者と締結した契約に係るものを除く．）

13）その他本研究に関連する文書又は記録

## 16.1. 試料・情報の二次利用について

保管されたDay0（介入製剤投与前）Day3およびDay7の血漿・全血・糞便に関して脂質代謝およびタンパク質代謝に関する解析を追加して行い，重症病態における下痢が栄養代謝に与える影響を解析する．

本研究に関わる研究者が本研究で得られた情報及び研究データを異なる研究目的で使用する可能性又は他の研究機関に提供する可能性があるが，その場合は，新たに研究計画書を作成し，当該研究に意見を聴くべき倫理審査委員会で承認を得た後に実施する．インフォームド・コンセントは，研究の内容にあわせて適切な方法で行う．

## 16.2. 試料・情報のバイオバンクとしての利用

本研究において該当なし．

# **17. 研究の資金源等，研究に係る利益相反管理**

## 17.1. 資金源及び財政上の関係

本研究は，株式会社大塚製薬工場から共同臨床研究契約により資金提供,及び試験品（ハイネックスリニュート）の提供を受けて実施する．株式会社大塚製薬工場は，試験品に関する情報は提供するが，試験の実施，解析，報告に係わることはない．

## 17.2. 利益相反管理

17.2.1.研究責任医師（又は研究代表医師）の利益相反管理

本臨床研究は，株式会社大塚製薬工場からの研究資金を受けて実施され，研究で使用する試験品であるハイネックスリニュートは，ハネックスリュートの製造販売業者である株式会社大塚製薬工場より無償提供を受ける．資金，試験品の提供を受けるにあたり，臨床研究法及び関連する法規に定める事項について，契約を締結している．研究代表医師は，利益相反管理基準を定め，実施医療機関の管理者による事実確認を行い，利益相反管理計画を作成する．研究代表医師は，利益相反管理基準及び利益相反管理計画について，実施計画に記載のある認定臨床研究審査委員会の意見を聴き，適切な管理を行う．

17.2.2.研究分担医師等の利益相反管理

本研究において，利益相反申告者となる研究分担医師及び統計解析責任者は，実施医療機関の管理者に事実関係の確認を行い，利益相反確認報告書を入手する．研究責任医師は，当該利益相反確認報告書を受け取りその内容を踏まえ，利益相反管理計画を作成し，実施計画に記載のある認定臨床研究審査委員会の意見を聴き，適切な管理を行う．

# **18.　研究対象者の費用負担・謝礼について**

本研究の実施にかかる費用のうち，個々の患者に要する医療費（診察費，入院費用，薬剤費，検査代など）については，本研究で行う治療がすべて保険診療範囲であることから自己負担分を研究対象者が支払う．本研究へ参加することで研究対象者に別途金銭的負担が増えることはない．本研究では，研究対象者に対して金銭的およびそれ以外による参加謝礼並びに負担軽減費等の支払い等はしない．試験品となるハイネックスリニュートは株式会社大塚製薬工場から提供を受け,グルセルナ-REXは研究代表医師が提供するため,研究対象者の負担はない..

# **19. 健康被害に対する補償**

本研究の参加に起因して研究対象者に健康被害が生じた場合，研究責任医師及び研究分担医師は，適切な治療及びその他必要な措置を行う．この場合の治療等は保険診療として行い，自己負担分の医療費を研究対象者が支払う．また，医療費・医療手当などの支給はない．

なお，本研究に起因する健康被害による賠償責任が生じた場合及び研究対象者に死亡又は後遺障害1　～3級の健康被害が生じた場合の補償に備えて，臨床研究賠償責任保険に加入する．補償には一定の条件があり，以下の事項を確認した場合は，補償に支払いを除外又は制限する場合がある．

（1） 研究計画書からの著しい逸脱があった場合

（2） 研究責任医師や研究分担医師等に故意又は過失，もしくは医療過誤があった場合

（3） 第三者の違法行為又は不履行があった場合

（4） 研究対象者に故意又は重大な過失があった場合　など

# **20. 定期報告**

## 20.1. 認定臨床研究審査委員会への定期報告

研究代表医師は，特定臨床研究の実施状況について，実施計画を厚生労働大臣に提出した日から起算して１年ごとに（当該期間満了後2月以内），実施医療機関の管理者に報告した上で，当該実施計画に記載された認定臨床研究審査委員会に定期報告を行う．実施状況の報告事項は以下のものとする．

(1) 参加した臨床研究対象者の数

(2) 疾病等の発生状況及びその後の経過

(3)　不適合の発生状況及びその後の対応

(4) 安全性及び科学的妥当性についての評価

(5)　利益相反管理基準に定める医薬品等製造販売業者等の関与に関する事項

研究代表医師は，認定臨床研究審査委員会に報告を行ったときは，その旨を，速やかに他の研究　責任医師に情報提供を行う．当該他の研究責任医師は，速やかに当該情報提供の内容を他の実施医療機関の管理者に報告する．

## 20.2. 厚生労働大臣への定期報告

（例文）

研究責任医師（又は研究代表医師）は，特定臨床研究の実施状況について，実施計画に記載された認定臨床研究審査委員会が意見を述べた日から起算して１月以内に，以下の事項について厚生労働大臣に報告する．

(1) 実施計画に記載されている認定臨床研究審査委員会の名称

(2) 認定臨床研究審査委員会による当該特定臨床研究の継続の適否

(3) 特定臨床研究に参加した特定臨床研究対象者の数

# **21. 研究の情報公開及び結果公表**

## 21.1. 研究の登録

本研究の実施に先立ち，厚生労働省が整備するデータベース（jRCT = Japan Registry of Clinical Trials）に記録（登録）する．研究計画書の変更及び研究の進捗に応じて適宜更新する．研究を終了したときは，研究の結果を登録する．

## 21.2. 研究結果の公表

研究代表医師は，主要評価項目報告書又は総括報告書及びその概要を作成する．その作成期限は，主たる評価項目又は全て評価項目に係るデータの収集を行うための期間が終了してから１年以内とする．

研究代表医師は，主要評価項目報告書又は総括報告書及びその概要を作成したとき，実施計画に記載された認定臨床研究審査委員会が意見を聴き，遅滞なく，実施医療機関の管理者に提出するとともに，主要評価項目報告書又は総括報告書の概要をjRCTに記録（登録）する．遅滞なくとは，当該委員会が意見を述べた日から起算して１月以内とする．研究代表医師は，実施医療機関の管理者への提出時に，その旨を他の研究責任医師に情報提供し，当該他の研究責任医師は，速やかに，当該情報提供の内容を他の実施医療機関の管理者に報告する．

## 21.3. 学会等の公表

本研究から得られた成果について，速やかに学会発表あるいは論文投稿による公表を行う．公表する際は，研究対象者等及びその関係者の人権又は研究者等及びその関係者の権利利益の保護のために必要な措置を講じた上で公表する．学会発表者及び論文の筆頭著者は協議の上決定し，発表者及び筆頭著者は，発表前及び投稿前に，研究代表医師の審査・承認を受ける．

# **22. 品質管理及び品質保証**

## 22.1. モニタリング

　研究代表医師は，モニタリングを実施する場合，次に掲げる事項について実施する

1. 研究対象者の人権の保護,安全の確保が図られていること
2. 臨床研究が最新の実施計画,研究計画書及び省令を遵守して実施されていること
3. 臨床研究の実施について研究対象者から文書により同意を得ていること
4. 記録等が正確であることについて原資料等に照らして検証すること

本試験では，データセンターに収集されるCRFのデータに基づいて中央モニタリングを行う．原資料とCRFとの照合を行う訪問モニタリングは実施しない．ただし，研究代表医師の要請があった場合は，On-siteモニタリングを実施することができる．モニタリング担当者は年に2回，研究代表医師の依頼により中央モニタリングを実施する．作成した定期モニタリングレポートは，研究代表医師に提出，検討され，指摘された問題点を各研究機関の研究者と情報共有し，その改善に努める．

## 22.2. 監査

本研究では，監査を実施しない．

# **23．研究成果の帰属（知的財産権）**

本研究の結果として，特許検討の知的財産権が生じた場合，その権利は公立大学法人横浜市立大学に帰属する．

# **24. 研究の実施体制**

## 24.1. 研究責任医師（又は研究代表医師）

研究機関名：横浜市立大学附属病院　集中治療部

研究機関住所:神奈川県横浜市金沢区福浦3-9

研究機関TEL：　045-787-2918

研究代表者名：中村謙介

研究代表者所属： 集中治療部

役割：研究の統括，遂行

## 24.2. 研究事務局

氏名：横山暢幸

所属：横浜市立大学附属病院　集中治療部

住所：〒236-0004　神奈川県横浜市金沢区福浦3-9

TEL：045-787-2918

役割：研究の遂行，各共同研究機関との連絡調整

## 24.3. 実施医療機関及び研究責任医師

表24.3 実施医療機関及び研究責任医師一覧

　　　　役割：患者登録，プロトコル治療の実施

| 実施医療機関 | 所属 | 職名 | 研究責任医師 |
| --- | --- | --- | --- |
| 横浜市立大学附属病院  〒236-0004　神奈川県横浜市金沢区福浦3-9  045-787-2918 | 集中治療部 | 医師 | 中村　謙介 |
| 慶應義塾大学病院  〒160-0016 東京都新宿区信濃町３５  03-3353-1211 | 救急科 | 医師 | 佐々木　淳一 |
| 聖マリアンナ医科大学  〒216-8511 神奈川県川崎市宮前区菅生２丁目１６−１  044-977-8111 | 救急医学 | 医師 | 永冨　彰仁 |
| 聖マリアンナ医科大学　横浜市西部病院  〒241-0811 神奈川県横浜市旭区矢指町1197-1  045-366-1111 | 救命救急センター | 医師 | 吉田　稔 |
| 日立総合病院  〒317-0077 茨城県日立市城南町２丁目１−１  0294-23-1111 | 救急集中治療科 | 医師 | 高橋　雄治 |
| 大阪医科薬科大学  〒569-8686　大阪府高槻市大学町2番7号  072-683-1221 | 救急医学教室 | 医師 | 畠山　淳司 |

## 24.4. データマネージメント責任者

氏名：柏木静

所属：横浜市立大学附属病院　集中治療部

住所：〒236-0004　神奈川県横浜市金沢区福浦3-9

TEL：045-787-2918

## 24.5. 統計解析責任者

篠田　覚

所属：横浜市立大学　医学部　臨床統計学教室

所在地：〒236-0004 神奈川県横浜市金沢区福浦3-9

電話番号：TEL：045-787-2572

## 24.6. モニタリングに関する責任者

氏名：柏木静

所属：横浜市立大学附属病院　集中治療部

住所：〒236-0004　神奈川県横浜市金沢区福浦3-9

TEL：045-787-2918

## 24.7. 監査に関する責任者

該当なし

## 24.8. 研究・開発計画支援担当者

該当なし

## 24.9. 調整管理実務担当者

該当なし

## 24.10.研究代表医師以外に研究を総括する者

該当なし

## 24.11. その他臨床研究に関連する臨床検査施設並びに医学的及び技術的部門・機関

該当なし

## 24.12. 外部委託機関

本研究に関する電子データベース構築，ランダム化割付業務及びアカウント発行業務は，TXP Medical株式会社に委託し,実施する．委託業務の実施状況等は委受託契約書に基づいて監督する．

機関名：TXP Medical株式会社

住所：〒101-0042 東京都千代田区神田東松下町４１−１ H¹O 神田 706

委託する業務の内容：電子データベース構築，ランダム化割付,アカウント発行業務

責任者：園生 智弘

# **25. 文献**

1) Reintam Blaser A, Starkopf J, Alhazzani W, et al. (2017) Early enteral nutrition in critically ill patients: ESCIM clinical practice guidelines. Intensive Care Med ;43:380e98

2) Singer, P., Reintam, A., Berger, M. M., et al. (2019) ESPEN Guideline ESPEN guideline on clinical nutrition in the intensive care unit. Clinical Nutrition, 38(1), 48–79

3) McClave, S. A., Taylor, B. E., Martindale, R. G., et al. (2016). Guidelines for the Provision and Assessment of Nutrition Support Therapy in the Adult Critically Ill Patient: Society of Critical Care Medicine (SCCM) and American Society for Parenteral and Enteral Nutrition (A.S.P.E.N.). Journal of Parenteral and Enteral Nutrition, 40(2), 159–211

4) Reintam A, Parm P, Kitus R, et al. (2009) Gastrointestinal symptoms in intensive care patients. Acta Anaesthesiol Scand.; 53:318-24

5) McClave SA, Sexton LK, Spain DA, et al. (1999) Enteral tube feeding in the intensive care unit: factors impeding adequate delivery. Crit Care Med; 27: 1252–1256.

6) Montejo JC. (1999) Enteral nutrition-related gastrointestinal complications in critically ill patients: a multicenter study. The Nutritional and Metabolic Working Group of the Spanish Society of Intensive Care Medicine and Coronary Units. Crit Care Med; 27: 1447–1453.

7) Reintam Blaser A, Poeze M, Malbrain M, et al. (2013) Gastrointestinal symptoms during the first week of intensive care are associated with poor outcome: a prospective multicentre study. Intensive Care Med; 39: 899–909.

8) McRorie, J. W., & McKeown, N. M. (2017) Understanding the Physics of Functional Fibers in the Gastrointestinal Tract: An Evidence-Based Approach to Resolving Enduring Misconceptions about Insoluble and Soluble Fiber. Journal of the Academy of Nutrition and Dietetics, 117(2), 251–264

9） Kazuo Hino, Sho Miyatake, Fumiyo Yamada, Naoyuki Endo, Ryosuke Akiyama, Goro Ebisu . Undigested low-methoxy pectin prevents diarrhea and induces colonic contraction during liquid-diet feeding in rats. Nutrition 78 (2020) 110804

10) Nakamura, K., Inokuchi, R., Fukushima, K., et al. (2019) Pectin-containing liquid enteral nutrition for critical care: a historical control and propensity score matched study. Asia Pacific Journal of Clinical Nutrition, 28(1), 57–63

11) Dionne, J. C., Mbuagbaw, L. (2023). Diarrhea in the critically ill: definitions, epidemiology, risk factors and outcomes. In Current Opinion in Critical Care, Vol. 29, Issue 2, pp. 138–144

12)Lewis, S. J., & Heaton, K. W. (1997). Stool form scale as a useful guide to intestinal transit time. Scandinavian Journal of Gastroenterology, 32(9), 920–924.

13) Vincent JL, Moreno R, Takala J, et al. (1996). The SOFA (Sepsis-related Organ Failure Assessment) score to describe organ dysfunction/failure. On behalf of the Working Group on Sepsis-Related Problems of the European Society of Intensive Care Medicine. Intensive Care Med. Jul;22(7):707-10. doi: 10.1007/BF01709751.

.

15) Brisard, L., Le Gouge, A., Lascarrou, J. B., et al. (2014). Impact of early enteral versus parenteral nutrition on mortality in patients requiring mechanical ventilation and catecholamines: Study protocol for a randomized controlled trial (NUTRIREA-2). Trials, 15(1).

16) F abregas N, Ewig S, Torres A, et al. (1999). Clinical diagnosis of ventilator associated pneumonia revisited: comparativevalidation using immediate post-mortem lung biopsies. Thorax ;54(10):867e73

17) Reignier, J., Boisramé-Helms, J., Brisard, L., Lascarrou, et al. (2018). Enteral versus parenteral early nutrition in ventilated adults with shock: a randomised, controlled, multicentre, open-label, parallel-group study (NUTRIREA-2). The Lancet, 391(10116), 133–143

E1) Taito S, Kawai Y, Liu K,etal. (2019) Diarrhea and patient outcomes in the intensivecare unit: Systematic review and meta-analysis. J Crit Care; 53:142–148

E2）Dionne, J. C., & Mbuagbaw, L. (2023). Diarrhea in the critically ill: definitions, epidemiology, risk factors and outcomes. Current Opinion in Critical Care (Vol. 29, Issue 2, pp. 138–144)

E3）Dionne JC, Mbuagbaw L, Devlin JW,etal. (2022) Diarrhea during critical illness: amulticenter cohort study. Intensive Care Med; 48:570–579.

E4) Dionne JC, Campbell T, Janisse N,etal .(2020) Mo1960 effect of fiber, osmolarity,and protein content of enteral nutrition on the development of diarrhea incritical illness. Gastroenterology; 158:S–994.

E5) Jordan, E. A., Moore, S. C. (2020). Enteral nutrition in critically ill adults: Literature review of protocols. In Nursing in Critical Care (Vol. 25, Issue 1, pp. 24–30).

# **26. 付録**

該当なし
